# Fungi from *Malus* in Qujing, China: two new species, three new records, and insights into potential host jumping and lifestyle switching

**DOI:** 10.3389/fcimb.2025.1517908

**Published:** 2025-03-11

**Authors:** Gui-Qing Zhang, Zhu-Mei Li, Xin-Lei Fan, Qi-Rui Li, Jaturong Kumla, Nakarin Suwannarach, Abdallah M. Elgorban, Ihab M. Moussa, Dong-Qin Dai, Nalin N. Wijayawardene

**Affiliations:** ^1^ Center for Yunnan Plateau Biological Resources Protection and Utilization, College of Biology and Food Engineering, Qujing Normal University, Qujing, China; ^2^ Department of Biology, Faculty of Science, Chiang Mai University, Chiang Mai, Thailand; ^3^ Center of Excellence in Microbial Diversity and Sustainable Utilization, Chiang Mai University, Chiang Mai, Thailand; ^4^ The Key Laboratory for Silviculture and Conservation of the Ministry of Education, Beijing Forestry University, Beijing, China; ^5^ Engineering Research Center of Southwest Bio-Pharmaceutical Resources, Ministry of Education, Guizhou University, Guiyang, Guizhou, China; ^6^ The High Efficacy Application of Natural Medicinal Resources Engineering Centre of Guizhou Province (The Key Laboratory of Optimal Utilization of Natural Medicine Resources), School of Pharmaceutical Sciences, Guizhou Medical University, Gui’an, Guizhou, China; ^7^ Center of Excellence in Biotechnology Research, King Saud University, Riyadh, Saudi Arabia; ^8^ Department of Botany and Microbiology, College of Science, King Saud University, Riyadh, Saudi Arabia; ^9^ Tropical Microbiology Research Foundation, Colombo, Sri Lanka

**Keywords:** apple tree, fungal diversity, life mode, phylogeny, taxonomy, two novel species

## Abstract

Apple trees [*Malus domestica* Borkh. (*Rosaceae*)] are one of the important temperate fruit crops in China. In comparison to other temperate fruits, such as grapes and pears, fungal studies (in Yunnan) associated with *M. domestica* are fewer in number. In the present study, we investigated fungi associated with *M. domestica* in Qujing City, Yunnan Province, China. Samples were collected from apple gardens in different locations. Single spore isolation was carried out to isolate saprobic fungi, while the surface sterilization method was carried out to isolate endophytic fungi. Molecular analyses were carried out to determine the phylogenetic placement of the new collections. Based on the combined methods of morphology and phylogeny, *Cytospora qujingensis* sp. nov. and *Hypoxylon malongense* sp. nov. are introduced as novel saprobic and endophytic taxa, respectively. Moreover, *Aureobasidium pullulans* and *Cytospora schulzeri* are reported as new geological records from southwestern China. *Allocryptovalsa castaneae* is reported on *M. domestica* in China for the first time. The checklist of fungi associated with *M. domestica* in China is presented.

## 1 Introduction

Yunnan Province, located in the interior low-latitude plateau in southwestern China, features a complex geography and unique climate. These attributes, in conjunction with its favorable ecological condition, contribute to the high quality of fruit yields in the region (Dong et al., 2023). Yunnan has a diverse range of fruits, which hosts 287 fruit plant species belonging to 118 genera and 49 families from tropical to temperate species, ranking first in China (Zhao et al., 2018). According to the Department of Agriculture and Rural Affairs of Yunnan Province (2021) (accessed 01 April 2024), over 11 million tons of fruits (mostly blueberries, strawberries, raspberries, oranges, and apples) were harvested in 2020.

Apple is one of the four major fruits in the world, rich in minerals and vitamins, with high nutritional value (Sachini et al., 2020). However, large amounts of chemical fertilizers are applied to increase productivity. Extensive use of chemicals causes serious environmental impacts. Farmers are fond of using fungicides since fruits are susceptible to diseases caused by fungal pathogens. *Cytospora* canker is one of the most widespread canker diseases of *Malus domestica* (Wang et al., 2024a). For example, 1) *Cytospora mali* causes severe necrosis of the branches and stems globally (Wang et al., 2007), and 2) *Cytospora parasitica* is reported to cause canker on apples in China (Ma et al., 2018).

Fungi are diverse in terms of shape, color, lifestyle, and distribution throughout any ecosystem (Bhunjun et al., 2022). Researchers estimated the diversity of fungal species ranging from half a million (May, 2000) to more than 12.5 million (Wu et al., 2019), even reaching 19.35 million (Tedersoo et al., 2021). Hitherto, Species Fungorum (2024) (accession date: 04 September 2024) lists all accepted species of fungi, currently 161,348 species, with only approximately 40,000 named fungal species in GenBank (Hawksworth and Lücking, 2017). An accurate estimate of the number of fungi will provide a better understanding of fungal diversity and biogeography (Hawksworth, 1991; Bhunjun et al., 2022). In traditional taxonomy, species amount was provided based on the host or morphological characters (sexual morph: ascomata, pseudoparaphyses, asci, ascospores; asexual morph: conidiomata, ostioles, conidiophores, conidiogenous cells, conidia). Meanwhile, with the application of DNA sequencing technologies, the reliability of introducing species based on morphological characters was questioned by taxonomists (Wijayawardene et al., 2021b). A vague “dark taxa” poses challenges to researchers in estimating fungal populations; therefore, it is more important to use different molecular approaches to accurately delineate species and to estimate species diversity (Cai et al., 2009; Wijayawardene et al., 2023), such as DNA barcoding (Seifert, 2009), environmental DNA (eDNA) metabarcoding (Tedersoo et al., 2014), metagenomics (Tedersoo et al., 2018), genome sequencing (Baldrian et al., 2022), and phylogenomic analysis (Wijayawardene et al., 2023). Estimating species numbers is critical for species conservation and ecosystem management since changes in fungal diversity can influence ecosystem performance (Genevieve et al., 2019). Our understanding of host specificity is unclear, particularly as new plant species are found and several habitats remain unexplored. Moreover, the knowledge about lifestyle switching between and within species remains unclear and thus needs extensive studies. Furthermore, the possibility of revealing novel fungal species and new host or country records creates more opportunities to discover new compounds, such as antimicrobial agents and enzymes (Suetrong et al., 2023). Researchers demonstrated the importance of revealing novel fungal species from different habitats and substrates, as well as reports of new host or country records. This allows us to enhance our understanding of lifestyle switching of one species in different hosts or the same host in different geographic regions and whether the same species produces new compounds to sustain an individual’s existence when it survives in different habitats, such as from the stratosphere (Wainwright et al., 2003) to the bottom of the Dead Sea (Oren and Gunde-Cimerman, 2012), from the Antarctic glaciers (Freeman et al., 2009) to torrid deserts (Gonçalves et al., 2016), and from the gut of flies (Blackwell, 2017) to deep oceanic sediments (Nagahama et al., 2011), and prevents the occurrence of fungal diseases. For example, 1) *Alternaria alternata* is a widespread fungus that is regularly isolated from plants as both endophyte and pathogen and in the soil as a saprophyte (Thomma, 2003; DeMers, 2022); and 2) *Aureobasidium pullulans* is one of the well-adapted saprophytes in the phyllosphere (McCormack et al., 1995; Dik and Elad, 1999) and postharvest diseases (Ippolito and Nigro, 2000) and also reported as an endophyte in symptomless plant organs (Loi et al., 2000).

In previous studies, more than 200 fungal species (including new species and known species) were reported on *M. domestica* from China (**Supplementary Table S1**). *Alternaria* spp. and *Penicillium* spp. are the most common pathogens associated with the diseases reported from apples (Basson et al., 2019). *Cytospora* is considered one of the most important pathogenic genera, which can cause canker and dieback disease in apple-producing areas in China, such as Gansu, Shanxi, Liaoning, and Yunnan provinces (Gui et al., 2015). During our field surveys in Qujing City (from January to September 2021), significant disease symptoms on fruits, leaves, and stems have not been observed. Hence, we investigated whether these plants harbor any taxa (endophytes or saprobes) that have been reported as pathogens in previous studies. It is well-established that some species could change their lifestyle due to other factors, such as nutritional requirements and host specificity (Rai and Agarkar, 2016). Furthermore, it was predicted that well-studied host plants in less-studied geographical regions could harbor more novel taxa (Wijayawardene et al., 2022a, b).

In the present study, based on the morphological characteristics and DNA sequence-based phylogenetic analyses, we introduce two novel species, i.e., *Cytospora qujingensis* sp. nov. (as a saprobe) and *Hypoxylon malongense* sp. nov. (as an endophyte), respectively. *Aureobasidium pullulans* (as an endophyte) and *Allocryptovalsa castaneae* and *Cytospora schulzeri* (as saprobes) are reported as new geographical or host records in southwestern China.

## 2 Materials and methods

### 2.1 Sampling and fungal isolation

Sampling was conducted during the fall season, using a random sampling method. Specifically, healthy and dead leaves and leaf litter of *M. domestica* were collected to assess fungal presence across different plant tissues from different apple orchards in Qujing City, Yunnan Province, China. The orchards under study were managed by local farmers who applied fungicides regularly as part of their standard pest and disease control practices. All the samples were stored in polythene bags and brought to the microbiology laboratory at Qujing Normal University.

Single spore isolation (for saprobes) was conducted as described by Dai et al. (2017). Stromata or conidiomata were sectioned by hand using a razor blade. The hymenium containing ascospores or conidia mass was removed and placed on a drop of sterile water on a flamed concave microscope slide and separated by sterile needles. The spore suspension was placed drop by drop on potato dextrose agar (PDA) plates, each containing a standard concentration of 39 g/L PDA, and incubated overnight in incubators at 28°C. The germinated spores were photographed and then transferred to a new PDA plate. Mycelium grew within 2 weeks, and the hyphal were transferred to three new PDA plates to get the pure culture for DNA extraction.

The surface sterilization method (for endophytes) was performed following Senanayake et al. (2020) but with a slight change. Plant leaves were cleaned with distilled water and then air-dried for 1 min. The leaf surface was sterilized by soaking in 75% ethanol for 30 s and then 1% sodium hypochlorite for 60 s. Finally, it was rinsed in sterile demineralized distilled water three times. Presterilized forceps were used to transfer the cleaned leaves onto the PDA after they had been chopped into fragments (approximately 5 mm × 5 mm) using a sterile scalpel blade on a sterile glass Petri dish under aseptic conditions within a laminar flow hood to minimize contamination. As for overnight growth in incubators at 28°C, the PDA plates were checked regularly, and individual colonies were transferred to a new PDA plate until pure strains were available for DNA extraction experiments.

### 2.2 Morphology and preserving

Morphological characteristics were observed and studied using a Leica DM1000 microscope and photographed by differential interference contrast (DIC). A Leica S8AP0 stereomicroscope with an HDMI 200C camera (Leica Corporation, Germany) was used for examining fruiting bodies. An Olympus BX53 compound microscope (Olympus Corporation, Japan) with differential interference contrast (Olympus BX53 DIC compound microscope with an Olympus DP74 camera, Japan) was used to observe and photograph the morphological characteristics. Colonies on PDA were observed and photographed using a Leica S8AP0 stereomicroscope with an HDMI 200C camera under appropriate lighting conditions to capture detailed morphological characteristics. A razor blade was used to obtain thin sections of stromata and conidiomata by hand.

Specimens were preserved at the Mycological Herbarium of Zhongkai University of Agriculture and Engineering (MHZU). Cultures were deposited at the Zhongkai University of Agriculture and Engineering (ZHKUCC). Duplicates of the specimens and type cultures were deposited at the Herbarium of Guizhou Medical University, Guiyang, China (GMB), and Guizhou Medical University Culture Collection (GMBCC) in Guiyang, China, respectively.

### 2.3 Registration of novel taxa

Index Fungorum Registration Identifiers were obtained from Index Fungorum (2024) (https://www.indexfungorum.org).

### 2.4 DNA extraction, PCR amplification, and sequencing

Fresh mycelium was cultured on PDA in incubators at 28°C overnight for 15 days. The genomic DNA was extracted from fresh cultures according to the specifications of the Biospin Fungal Genomic DNA Extraction Kit (bioflux^®^). Each gene region was separately amplified using both forward and reverse primers, which are provided in **Table 1**. A final volume of polymerase chain reaction (PCR) was prepared at 25 μL, including 1 μL of DNA template, 1 μL of each forward and reverse primer, 12.5 μL of 2× Taq PCR Master Mix, and 9.5 μL of double-distilled water (ddH_2_O) as described by Dai et al. (2017). The PCR thermal cycling procedure for amplifying ITS, LSU, and *rpb*2 was conducted as explained by Ma et al. (2022), while *tef*1-α, *tub*, and *act* genes were conducted as explained by Jia et al. (2024), respectively. The PCR products were obtained in Shanghai Majorbio Bio-Pharm Technology Co., Ltd., and BGI Tech Solutions Co., Ltd. (BGI-Tech, China) for sequencing.

**Table 1:** Partial gene regions and primers used in this study.

Loci	PCR primers (forward/reverse)	References
ITS	ITS5/ITS4	White et al. (1990)
LSU	LR0R/LR5	Vilgalys and Hester (1990)
*rpb*2	fRPB2-5f/fRPB2-7cr	Liu et al. (1999)
*tef*1-α	EF1-728F/EF1-1567R	Rehner and Buckley (2005)
*tub*	T1/T22	O’Donnell and Cigelnik (1997)
	T1/Bt2b	Glass and Donaldson (1995)
*act*	ACT-512F/ACT-783R	Carbone and Kohn (1999)

### 2.5 Phylogenetic analyses

Closely related sequences were downloaded from GenBank based on blast similarity and recent publications (Fan et al., 2020; Maharachchikumbura et al., 2022; Wu et al., 2023; Zhang et al., 2024c) (**Supplementary Tables S2**–**S5**). Mafft v.7.215 (http://mafft.cbrc.jp/alignment/server/index.html) (Katoh and Standley, 2013) was used to align the single gene sequence, automatic cutting was done in Trimal.v1.2rev59, and the final improvements were performed in BioEdit v.7.0.5.2 (Hall, 2004) by hand. The combined gene regions of ITS, *tef*1-α, *rpb*2, *tub*, and *act* (*Cytospora*); ITS and *tub* (*Allocryptovalsa*); ITS and LSU (*Aureobasidium*); and ITS, LSU, *rpb*2, and *tub* (*Hypoxylon*) regions were performed in BioEdit v. 7.0.5.2 (Hall, 2004) manually. The combined datasets in FASTA format were converted to PHYLIP and NEXUS formats by using ALTER (Alignment Transformation Environment online, http://sing.ei.uvigo.es/ALTER/) (Glez-Peña et al., 2010). The online tool FindModel (http://www.hiv.lanl.gov/content/sequence/findmodel/findmodel.html) was used to determine the best nucleotide substitution model for each partition data (Dai et al., 2022). The phylogenetic trees were constructed by maximum likelihood (ML) and Bayesian analyses.

ML analysis was carried out via the online portal CIPRES Science Gateway v. 3.3 (Miller et al., 2010), using RAxML-HPC v.8 on XSEDE (8.2.12) tool, with the default settings but adapted with the GAMMA nucleotide substitution model and 1,000 rapid bootstrap replicates.

Bayesian analysis was carried out by MrBayes v. 3.0b4 (Ronquist and Huelsenbeck, 2003), and the model of evolution was estimated with MrModeltest v. 2.2 (Nylander, 2004). The posterior probabilities (PP) (Rannala and Yang, 1996; Zhaxybayeva and Gogarten, 2002) were determined by the following Markov chain Monte Carlo sampling (MCMC) in MrBayes v.3.0b4 (Huelsenbeck and Ronquist, 2001). Six simultaneous Markov chains were run for 1,000,000 generations, with trees sampled every 100th generation. The preburn was set to 0.25 and the run was automatically stopped when the mean standard deviation of the split frequency reached below 0.01 (Maharachchikumbura et al., 2015).

Trees were viewed by FigTree v. 1.4.0 (http://tree.bio.ed.ac.uk/software/figtree/) (Rambaut, 2006), and the phylogram was edited by Microsoft Office PowerPoint 2016 (Microsoft Inc., Redmond, WA, USA) and converted to jpg. file by the Adobe PhotoShop CC 2018 software (Jiang et al., 2021).

## 3 Results and discussion

### 3.1 Phylogenetic analyses

#### 3.1.1 Multigene analyses for *Cytospora* (*Valsaceae*, *Diaporthales*, and *Sordariomycetes*)

**Figure 1:**
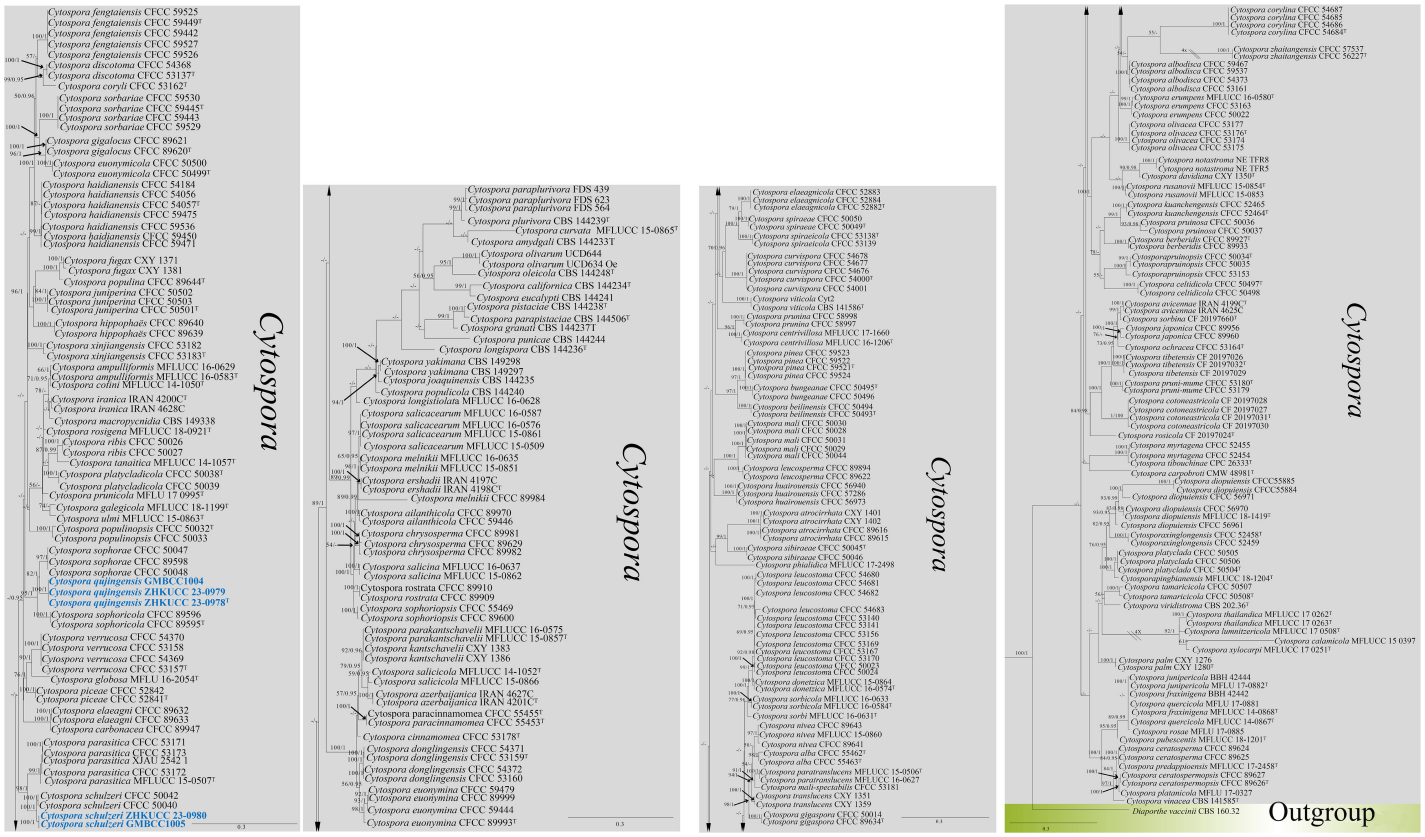
The phylogenetic tree from the best scoring of the RAxML analysis based on combined gene regions (ITS, *tef*1-α, *rpb*2, *tub*, and *act*) is rooted to *Diaporthe vaccinii* (CBS 160.32). Bootstrap values for maximum likelihood (MLBP) and Bayesian posterior probabilities (BYPP) equal to or greater than 50% and 0.95 are given at the respective branches. A hyphen (-) means a value lower than 50% (ML) or 0.95 (PP). New isolates are labeled in blue bold and ex-types are indicated in “T” (**Supplementary Table S2**).

The combined gene regions of ITS, *tef*1-α, *rpb*2, *tub*, and *act* contained 300 isolates, which comprised 2,249 characters with gaps. Single gene analysis was carried out to compare the topology of the tree and clade stability. *Diaporthe vaccinii* (CBS 160.32) was used as the outgroup taxon. The best-scoring RAxML tree with a final likelihood value of −53,458.778097 is presented in **Figure 1**. The matrix had 1,755 distinct alignment patterns, with 23.31% of undetermined characters or gaps. The estimated base frequencies were as follows: A = 0.243243, C = 0.273232, G = 0.242722, and T = 0.229822; the substitution rates were AC = 1.503463, AG = 3.421602, AT = 1.349798, CG = 1.091741, CT = 6.308732, and GT = 1.000000; and the gamma distribution shape parameter alpha = 0.483398. The GTR+I+G model was selected as the best model based on MrModeltest and was used for the Bayesian analysis.

In the phylogenetic analysis, our new isolates [ZHKUCC 23-0978 (ex-type), ZHKUCC 23-0979, and GMBCC1004] formed a monophyletic clade (82% ML, 1.00 PP) sister to *Cytospora sophoricola* [CFCC 89595 (ex-type) and CFCC 89596] and *C. sophorae* (CFCC 89598, CFCC 50048, and CFCC 50047). Hence, the taxon which is represented by ZHKUCC 23-0978, ZHKUCC 23-0979, and GMBCC1004 is introduced as a novel species in *Cytospora* s. str. as *C. qujingensis* (see taxonomy section for further details). Furthermore, two new strains, ZHKUCC 23-0980 and GMBCC1005, were grouped with *C. schulzeri* (CFCC 50040 and CFCC 50042) with high statistical values (100% ML, 1.00 PP). Based on phylogenetic analyses and morphological comparisons, we confirmed that these two strains represent *C. schulzeri* (see taxonomy section for morphological comparison).

#### 3.1.2 Multigene analyses for *Allocryptovalsa* (*Diatrypaceae, Xylariales*, and *Sordariomycetes*)

**Figure 2:**
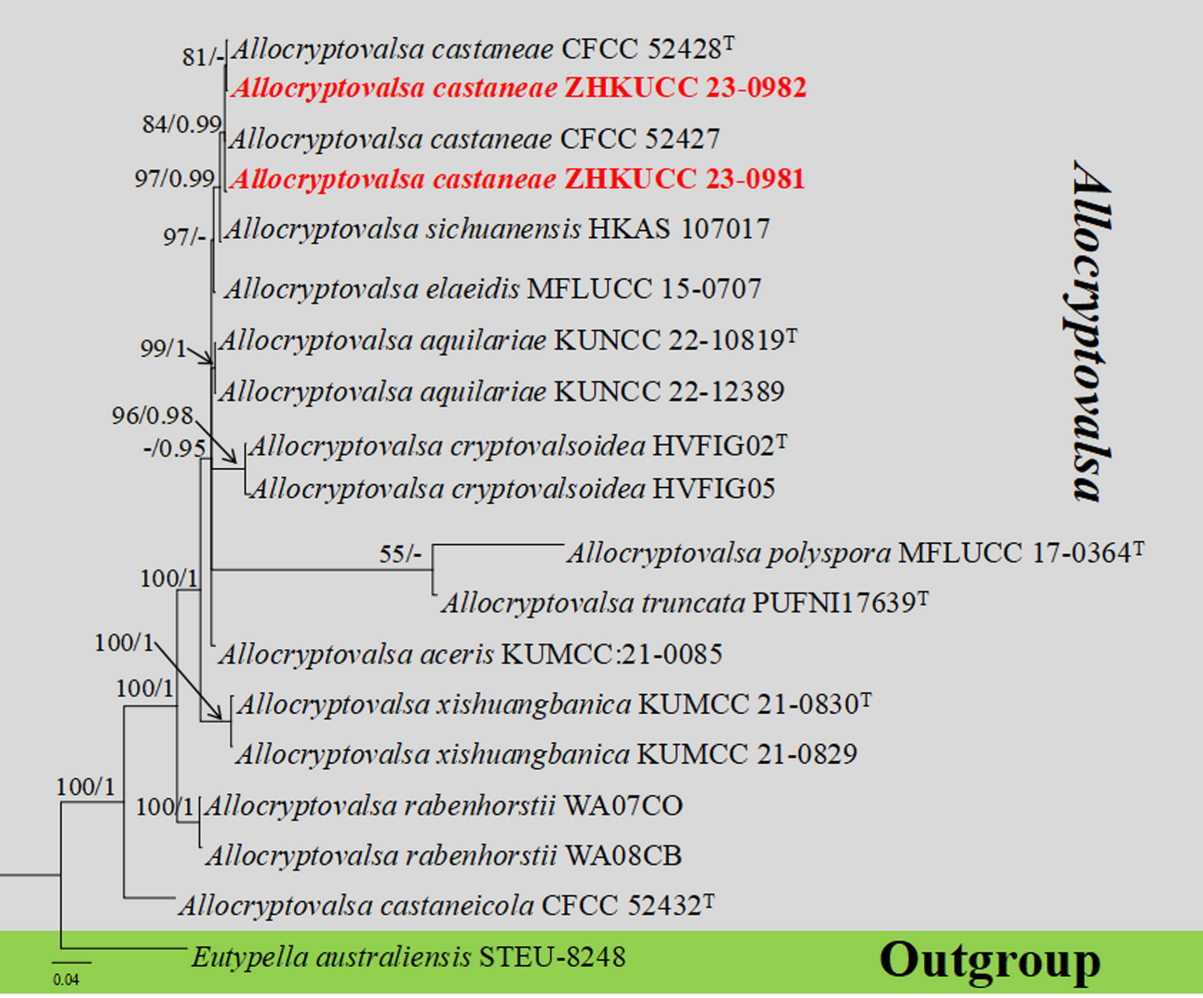
The phylogenetic tree from the best scoring of the RAxML analysis based on combined gene regions (ITS and *tub*) is rooted to *Eutypella australiensis* (STEU-8248). Bootstrap values for maximum likelihood (MLBP) and Bayesian posterior probabilities (BYPP) equal to or greater than 50% and 0.95 are given at the respective branches. A hyphen (-) means a value lower than 50% (ML) or 0.95 (PP). New isolates are labeled in red bold and ex-types are indicated in “T” (**Supplementary Table S3**).

The combined gene regions of ITS and *tub* contained 19 isolates, which comprised 1,221 characters with gaps. Single gene analysis was carried out to compare the topology of the tree and clade stability. *Eutypella australiensis* (STEU-8248) was used as the outgroup taxon. The best-scoring RAxML tree with a final likelihood value of 3,671.144837 is presented in **Figure 2**. The matrix had 287 distinct alignment patterns, with 25.59% of undetermined characters or gaps. The estimated base frequencies were as follows: A = 0.224076, C = 0.274997, G = 0.237980, and T = 0.262947; the substitution rates were AC = 1.266141, AG = 2.802018, AT = 1.131189, CG = 1.718083, CT = 4.262046, and GT = 1.000000; and the gamma distribution shape parameter alpha = 0.821838. The GTR+I+G model was selected as the best model based on MrModeltest and was used for the Bayesian analysis. In the phylogenetic tree, our two new strains of *Allocryptovalsa* (ZHKUCC 23-0981 and ZHKUCC 23-0982) were clustered with *Allocryptovalsa castaneae* [CFCC 52428 (ex-type) and CFCC 52427] with high statistical support (84% ML, 0.99 PP).

#### 3.1.3 Multigene analyses for *Aureobasidium* (*Saccotheciaceae*, *Dothideales*, and *Dothideomycetes*)

**Figure 3:**
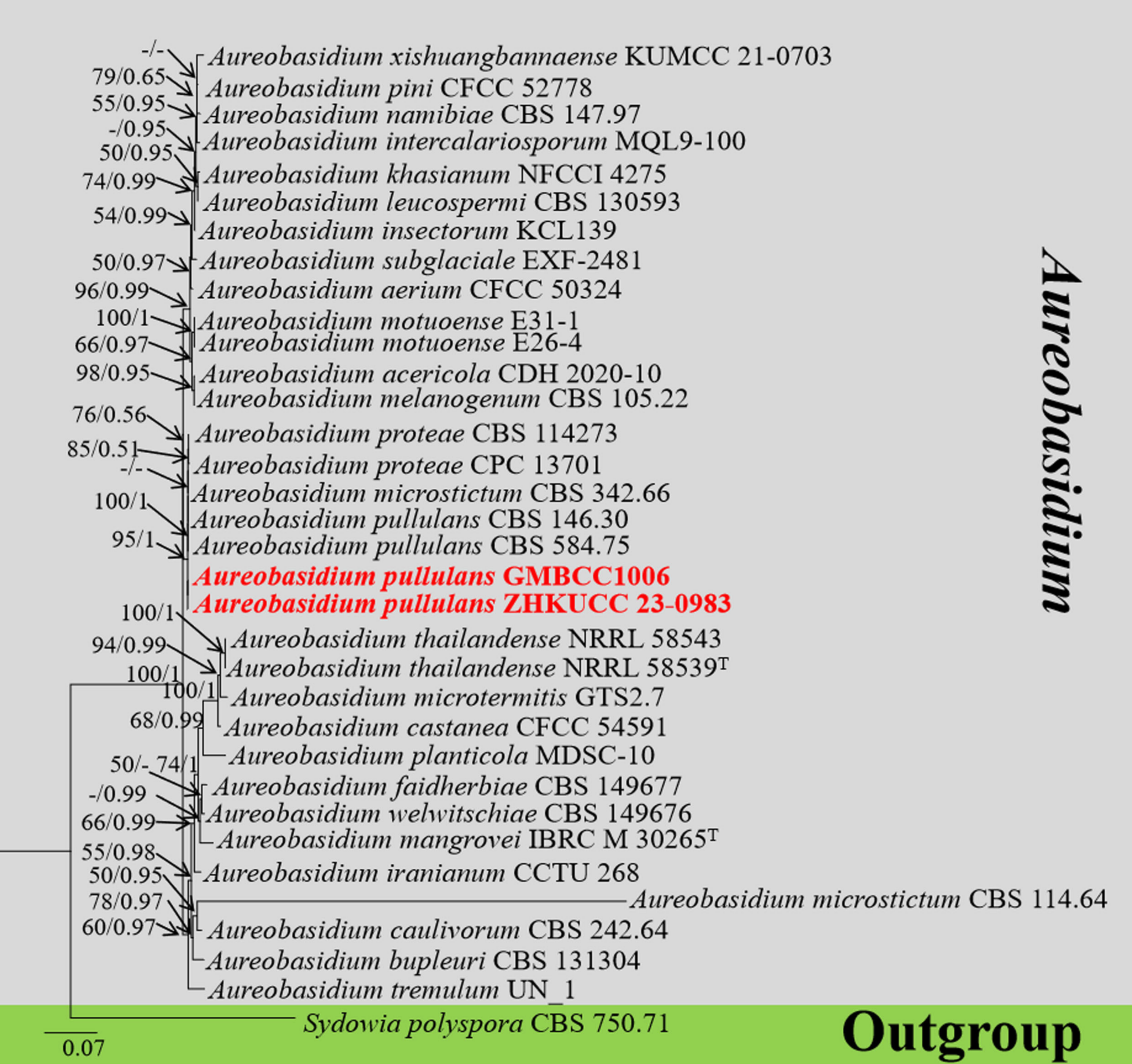
The phylogenetic tree from the best scoring of the RAxML analysis based on combined LSU and ITS is rooted to *Sydowia polyspora* (CBS 750.71). Bootstrap values for maximum likelihood (MLBP) and Bayesian posterior probabilities (BYPP) equal to or greater than 50% and 0.95 are given at the respective branches. A hyphen (-) means a value lower than 50% (ML) or 0.95 (PP). New isolates are labeled in red bold and ex-types are indicated in “T” (**Supplementary Table S4**).

The combined gene regions of LSU and ITS contained 34 isolates, which comprised 1,410 characters with gaps. Single gene analysis was carried out to compare the topology of the tree and clade stability. *Sydowia polyspora* (CBS 750.71) was used as the outgroup taxon. The best-scoring RAxML tree with a final likelihood value of −6,098.421647 is presented in **Figure 3**. The matrix had 298 distinct alignment patterns, with 21.36% of undetermined characters or gaps. The estimated base frequencies were as follows: A = 0.256565, C = 0.222028, G = 0.279511, and T = 0.241896; the substitution rates were AC = 1.507336, AG = 2.554163, AT = 1.625168, CG = 1.056662, CT = 4.675945, and GT = 1.000000; and the gamma distribution shape parameter alpha = 2.840594. The GTR+I+G model was selected as the best model based on MrModeltest and was used for the Bayesian analysis. In the phylogenetic tree, two newly generated strains of *Aureobasidium* (ZHKUCC 23-0983 and GMBCC1006) were clustered in the clade that comprises *Au. pullulans* (CBS 584.75 and CBS 146.30), *Au. proteae* (CBS 114273 and CPC13701), and *Au. microstictum* (CBS342.66) with high statistical values (91% ML, 1.00 PP). The placement of the abovementioned species agrees with Humphries et al. (2017) and Wu et al. (2023), who performed their analysis based on LSU and ITS gene regions. Thus, we compared the conidial morphologies of the new collection against the other three species and confirmed that our collection belongs to *Au. pullulans* (see taxonomic key in the taxonomy section).

#### 3.1.4 Multigene analyses for *Hypoxylon* (*Hypoxylaceae*, *Xylariales*, and *Sordariomycetes*)

**Figure 4:**
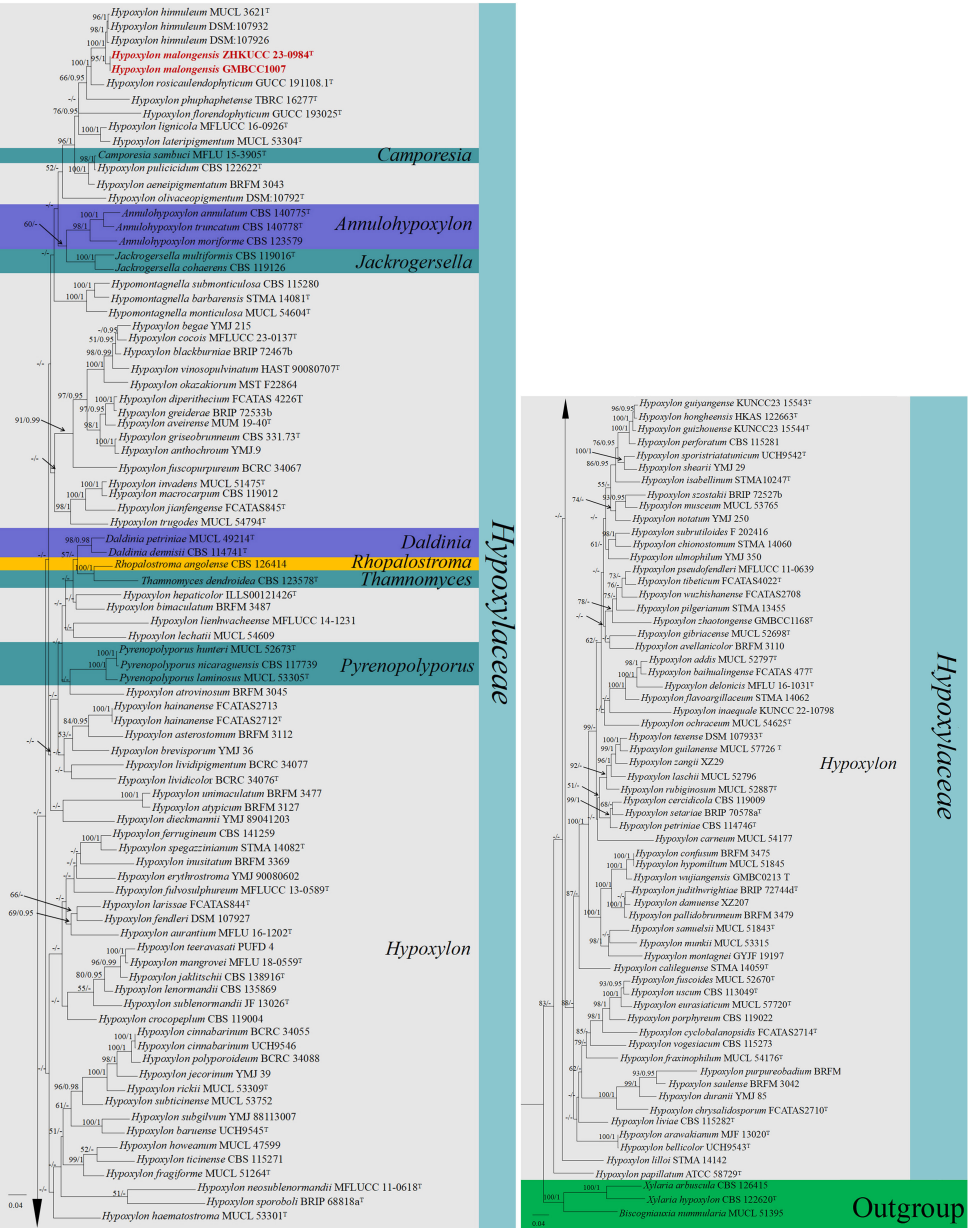
The phylogenetic tree from the best scoring of the RAxML analysis based on combined gene regions (ITS, LSU, *rpb*2, and *tub*) is rooted to *Xylaria arbuscula* (CBS 126415), *X. hypoxylon* (CBS 122620), and *Biscogniauxia nummularia* (MUCL 51395). Bootstrap values for maximum likelihood (MLBP) and Bayesian posterior probabilities (BYPP) equal to or greater than 50% and 0.95 are given at the respective branches. A hyphen (-) means a value lower than 50% (ML) or 0.95 (PP). New isolates are labeled in red bold and ex-types are indicated in “T” (**Supplementary Table S5**).

The combined gene regions of ITS, LSU, *rpb*2, and *tub* contained 150 strains in the sequence analysis, which comprise 3,728 characters with gaps. Single gene analysis was carried out and compared with each species, to compare the topology of the tree and clade stability. *Xylaria arbuscula* (CBS 126415), *X. hypoxylon* (CBS 122620), and *Biscogniauxia nummularia* (MUCL 51395) are set as the outgroup taxa. The best-scoring RAxML tree with a final likelihood value of −84,270.809729 is presented. The matrix had 2,144 distinct alignment patterns, with 33.59% of undetermined characters or gaps. The estimated base frequencies were as follows: A = 0.232029, C = 0.266223, G = 0.261601, and T = 0.240148; the substitution rates were AC = 1.022186, AG = 4.161361, AT = 1.213956, CG = 0.910957, CT = 5.597031, and GT = 1.000000; and the gamma distribution shape parameter alpha = 0.280100 (**Figure 4**). The GTR+I+G model was selected as the best model based on MrModeltest and was used for the Bayesian analysis. Overall tree topologies based on ML and Bayesian inference (BI) analyses were similar and not significantly different. In the phylogenetic analysis (**Figure 4**), our new collections [ZHKUCC 23-0984 (ex-type) and GMBCC1007] clustered in clade *Hypoxylon* and formed an independent lineage sister to *Hypoxylon hinnuleum* [MUCL 3621 (ex-type), DSM:107932, and DSM:107926] with relatively high statistical support (100% ML, 1.00 PP). Hence, we introduced *H. malongense* to accommodate our new collection from *M. domestica* (see taxonomy section for further details).

### 3.2 Taxonomy

***Cytospora*** Ehrenb., Sylv. mycol. berol. (Berlin): 28

Index Fungorum Registration Identifier: 7904

Type species: *Cytospora chrysosperma* (Pers.) Fr., Syst. mycol. (Lundae) 2(2): 542

*Cytospora* Ehrenb. was typified by *C. chrysosperma* (Pers.) Fr, and the members were reported as important plant pathogens, saprobes, and endophytes on branches and twigs of a broad range of plants with a worldwide distribution (Fan et al., 2020). Currently, 696 epithets of *Cytospora* have been listed in Index Fungorum (2024) (accessed 23 June 2024), but many species lack herbarium materials, ex-type, and molecular data. The asexual morph of *Cytospora* is characterized by pycnidial locules, single or labyrinthine, conidiophores filamentous, conidia hyaline and allantoid; however, the sexual morph shows clavate to elongate obovoid asci, with four or eight hyaline, allantoid ascospores (Spielman, 1983, 1985). In the traditional taxonomy, host-based methods, such as morphological characteristics of the host and shape and size of conidia, were mainly used to define the species of *Cytospora* (Fan et al., 2020). However, in the past two decades, morphological identification and phylogenetic species recognition concepts have led to the description of several additional new species of *Cytospora* (Lawrence et al., 2017, 2018; Fan et al., 2020). In this study, we collected two *Cytospora* species from apples. Morpho-molecular analyses confirmed that one of them is a novel taxon of *Cytospora*; thus, we introduced our collections [(ZHKUCC 23-0978 (ex-type), ZHKUCC 23-0979, and GMBCC1004] as a novel species, viz., *C. qujingensis* and collections (ZHKUCC 23-0980 and GMBCC1005) were confirmed as the first record of *C. schulzeri* on *M. domestica* from Yunnan Province, southwestern China.

***Cytospora qujingensis*** G.Q. Zhang, Wijayaw., & X.L. Fan, **sp. nov.**

Index Fungorum Registration Identifier: IF902662

**Figure 5**

**Figure 5:**
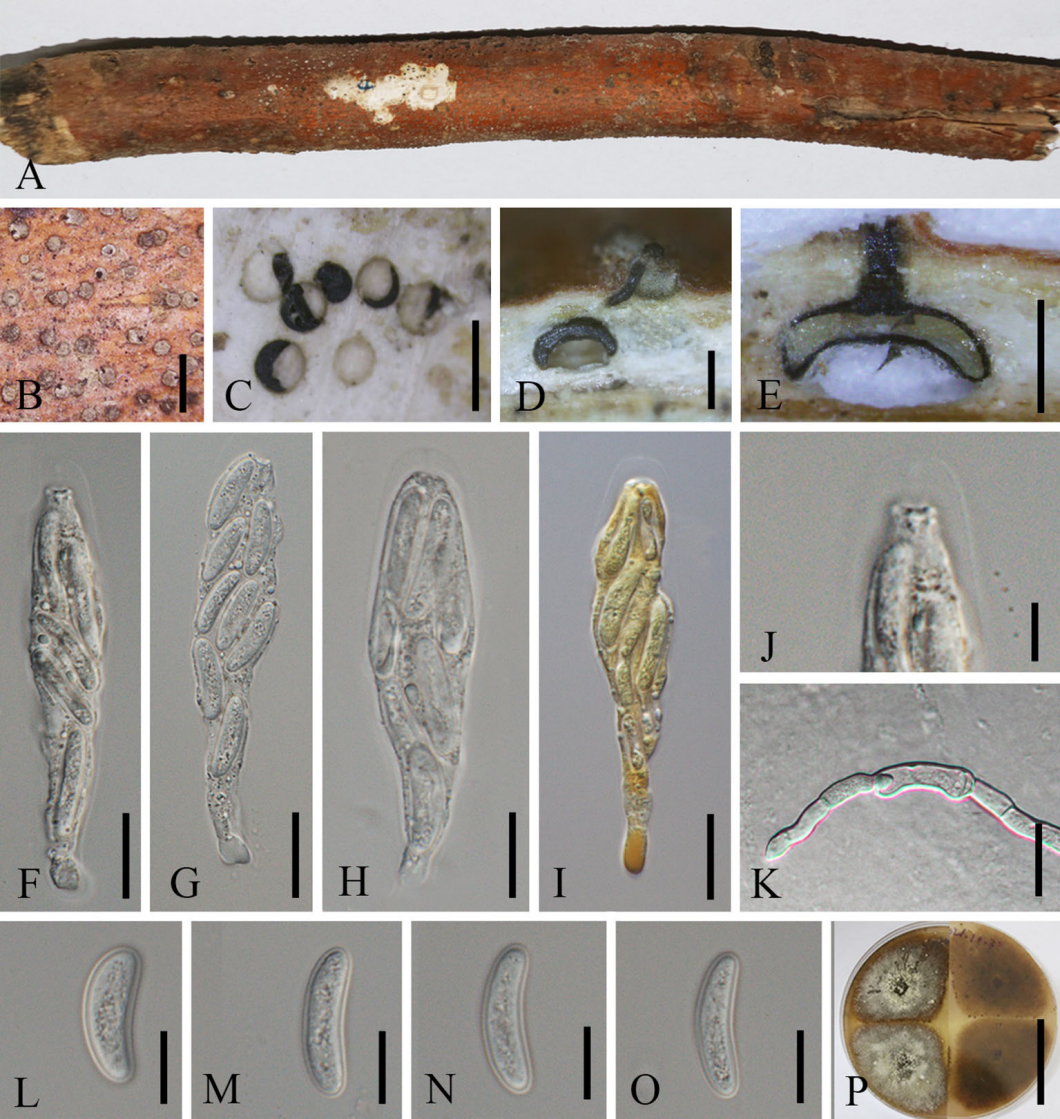
*Cytospora qujingensis* (MHZU 23-0265, holotype). **(A, B)** Habit of stromata on a branch. **(C)** Transverse section through stroma. **(D, E)** Longitudinal section through stroma. **(F–H)** Asci in water. **(I)** Asci in in Melzer’s reagent shows “J-” apical apparatus. **(J)** Apical apparatus of an ascus. **(K)** Germinating ascospore. **(L–O)** Ascospores. **(P)** Colonies on PDA from above and below. Scale bars: **(B)** = 1 mm, **(C–E)** = 0.5 mm, **(F–I)** = 15 μm, **(J–O)** = 5 μm, and **(P)** = 5 cm.

*Etymology*: Named after the location “Qujing” where the new taxon was first discovered.

*Diagnosis*: *Cytospora qujingensis* differs from other *Cytospora* species by its asci with “J-” apical ring and larger size of asci (60–85 × 10–16 µm) and ascospores (10–25 × 4–6 µm).

*Holotype*: MHZU 23-0265

*Description*: *Saprobic* on branches of *M. domestica* in China. **Sexual morph:** *Stromata* 390–550 μm in length and 160–330 μm in width (av. = 450 × 230 μm, *n* = 30), circular to ovoid, brown to black, usually scattered, initially immersed in the bark, slightly to strongly erumpent through the surface of bark when mature. *Conceptacle* absent. *Ectostromatic disc* inconspicuous, usually with a single ostiolar neck, 75–100 µm diam. *Ostioles* 140–170 µm diam., centric or lateral, slightly papillate, dark brown to black. *Perithecia* 420–530 × 285–300 μm (av. = 470 × 290 μm, *n* = 5), solitary, flask-shaped to spherical, black, arranged circularly. *Paraphyses* may be lacking at maturity but usually present, often collapsed and broad. *Asci*, 60–85 × 10–16 μm (av. = 70 × 12.5 μm, *n* = 10), eight-spored, unitunicate, clavate to elongate, with an indistinct pedicel, apically rounded and thinned, “J-” apical ring in Melzer’s reagent, refractive, refractive truncated at the top. *Ascospores* 10–25 × 4–6 μm (av. = 15 × 4.5 μm, *n* = 25), biseriate to overlapping, or irregularly arranged, elongate-allantoid, straight to slightly curved, slightly constricted at both ends, normally rounded, hyaline, smooth, thin-walled. **Asexual morph**: not observed.

*Culture characteristics*: Ascospores germinating on PDA, producing germ tubes from both ends within 24 h. Colonies growing on PDA, reaching 6–9 cm diam. after 7 days at 28°C, white surface, growing up to 6 cm diam. with irregular margins, covering the 9-cm plate after 14 days. In reverse, the cultures are the same as the upper color after 3 days, becoming isabelline to umber after 7–14 days. Colonies are felty with a heterogeneous texture, lacking aerial mycelium.

*Material examined*: CHINA, Yunnan Province, Qujing City, 25°47′77″N, 62°34′96″E, on the dead branch of *M. domestica*, 1 October 2021, Guiqing Zhang, Z4 = MHZU 23-0265 (holotype), ex-type: ZHKUCC 23-0978; *Ibid*. Z4-1 = GMB1004 (isotype), ex-isotype: GMBCC 1004; *Ibid*. Z14 living culture: ZHKUCC 23-0979.

*Note*s: *Cytospora qujingensis* was collected as a saprobe on a dead branch of *M. domestica*. In the phylogenetic analysis, *C. qujingensis* grouped with *C. sophorae* and *C. sophoricola* (**Figure 1**), two asexually typified taxa (Fan et al., 2013). Among these two species, our new species shows a closer phylogenetic relationship with *C. sophorae* (CFCC 50047, CFCC 50048, and CFCC 89598), supported by 82% ML and 1.00 PP statistical support values (**Figure 1**). However, the representative strains of *C. sophorae* are not type strains but have been used in the phylogenetic analyses by Jia et al. (2024). The reference strains were isolated from *Styphnolobium japonicum* (*Fabaceae*) and *Magnolia grandiflora* (*Magnoliaceae*) in the Gansu and Shanxi provinces in China (Fan et al., 2020), whereas our new strains originate from *M. domestica* (*Rosaceae*) in Yunnan Province, southwestern China.

Comparative analysis of the base pairs of ITS, *te*f1-α, *rpb*2, *tub*, and *act* gene regions, our isolate [ZHKUCC 23-0978, (ex-type)] show significant differences with *C. sophorae* (CFCC 89598, CFCC 50048, and CFCC 50047): 3/489 bp (0.6%), 20/721 bp (2.8%), 23/260 bp (8.8%, including three gaps), 41/508 bp (8.1%, including six gaps), and 4/237 bp (1.7%), respectively. These phylogenetic incongruities, alongside ecological distinctions, form the basis for proposing that ZHKUCC 23-0978 (ex-type), ZHKUCC 23-0979, and GMBCC1004 belong to a new species, *Cytospora qujingensis*.

Morphologically, *C. qujingensis* shares similarities with type species *C. chrysosperma*, and *C. schulzeri* in terms of asci and ascospore characteristics. However, our isolates can distinguish *C. chrysosperma* and *C. schulzeri* (teleomorph *Valsa malicola*) by their remarkable features, which our isolates with a single perithecial stromata, asci with a “J-” apical ring, refractive, while *C. chrysosperma* characterized by 4–8 perithecia arranged circinately in black entostromata, and *C. schulzeri* with 5–14 perithecia, both are not noted as having an apical ring (Fan et al., 2015, 2020) (more details are shown in **Table 2**). Phylogenetically, both *C. chrysosperma* and *C. schulzeri* are not closely related to our new isolates. Recently, Wang et al. (2024a) introduced three novel species of *Cytospora*, viz. *C. hejingensis* R. Ma & Ning Jiang, *C. kunsensis* R. Ma & Ning Jiang, and *C. jilongensis* R. Ma & Ning Jiang, the first two of which are revealed for their sexual morph while the last one was reported as its asexual morph. However, these three species are not included in our phylogenetic tree. The phylogenetic tree in Wang et al. (2024a) shows that these three species are not closely related to our novel isolates and are accommodated in a separate clade. Furthermore, the morphology among them supported by our new isolates is different from them (**Table 2**). The inability to obtain the asexual morph of our new isolates under laboratory conditions is a limitation of our study. Future discovery of the sexual morphs of *C. sophorae* and *C. sophoricola* would provide insights into whether these species should be considered distinct or a single species. Until then, we recommend treating them as two distinct species.

**Table 2:** Diagnostic characteristics of *Cytospora* species.

Morphological and colony characters	Species name and references				
	*C. chrysosperma* (Fan et al., 2015)	*C. hejingensis* (Wang et al., 2024a)	*C. qujingensis* (This study)	*C. kunsensis* (Wang et al., 2024a)	*C. schulzeri* (Fan et al., 2020)
**Stromata**	With 4–8 perithecia arranged circinately, 0.86–1.19 mm diam.	With 4–9 perithecia arranged irregularly, 400–1,250 µm diam.	Single perithecia, 390–550 µm diam.	With 5–11 perithecia arranged circularly, 750–1,350 µm diam.	With 5–14 perithecia arranged circularly or irregularly, 980–1,600 µm diam.
**Ectostromatic disc and ostiole**	Ectostromatic disc was obscured by tightly ostiolar necks, 0.20–0.34 mm diam., ostioles numerous	Ectostromatic disc inconspicuous, surrounded by tightly aggregated ostiolar necks, 100–350 µm diam., ostioles numerous, concentrated, 35–90 µm diam.	Ectostromatic disc inconspicuous, with single ostiolar necks 75–100 µm diam., ostioles centric or lateral, slightly papillate, 140–170 µm diam.	Ectostromatic disc surrounded by tightly aggregated ostiolar necks, 100–350 µm diam., ostioles numerous, concentrated, 40–90 µm diam.	Ectostromatic disc surrounded by tightly ostiolar necks, 285–465 µm diam., ostioles numerous, concentrated, 56–122 µm diam.
**Perithecia**	Flask-shaped to spherical, 0.26–0.34 mm diam.	Spherical, 120–300 µm diam.	Flask-shaped to spherical, solitary, 420–530 µm diam.	Spherical, 180–420 µm diam.	Flask-shaped to spherical, 235–350 µm diam.
**Asci**	Asci clavate to elongate obovoid, 38.6–43.8 × 5.1–6.2 µm	Asci clavate, 38–77 × 7–12.5 µm	Asci clavate to elongate, 60–85 × 10–16 μm, with an indistinct pedicel, with “J-” apical ring in Melzer’s reagent	Asci clavate, 38–86 × 7.5–13.5 µm	Asci clavate to elongate obovoid, 22.5–49 × 4–10 μm
**Ascospores**	Elongate-allantoid, 8.3–13.1 × 2.0–2.8 µm	Biseriate, allantoid, 6.5–9 × 2–2.5 µm	Elongate-allantoid, straight to slightly curved, 10–25 × 4–6 μm	Biseriate, allantoid, 10–19.5 × 2–2.5 µm	Ascospores not observed

***Cytospora schulzeri*** Sacc. & P. Syd., Syll. fung. (Abellini) 14(2): 918

Index Fungorum Registration Identifier: 140461

**Figure 6**

**Figure 6:**
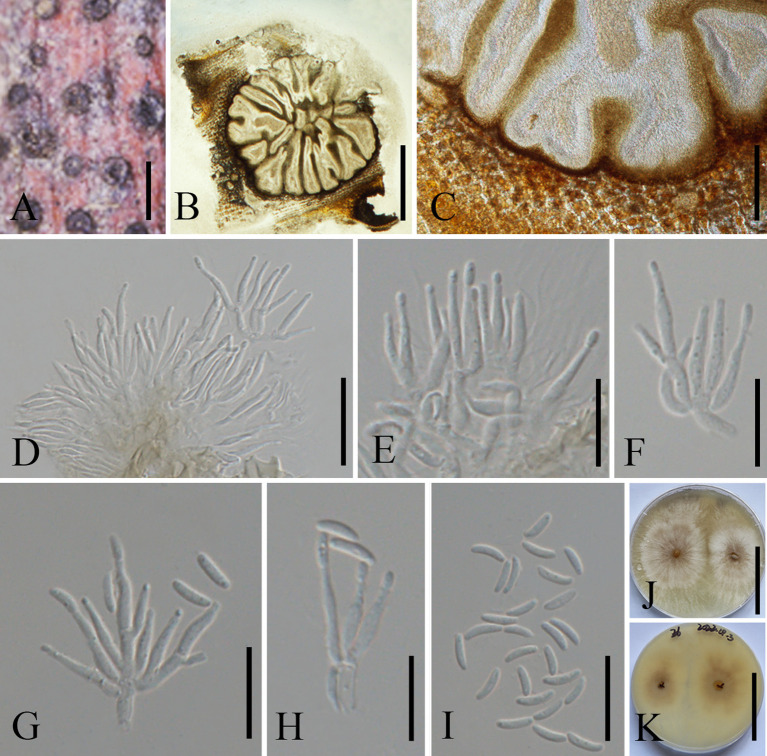
*Cytospora schulzeri* on a dead stem of *Malus domestica* (MHZU 23-0266). **(A)** Habit of conidiomata on a branch. **(B)** Transverse section through conidiomata. **(C)** Pycnidial wall. **(D–H)** Immature and mature conidia attached to conidiogenous cells. **(I)** Conidia. **(J, K)** Colonies on PDA from above and below (**J**: above, **K**: below). Scale bars: **(A)** = 300 μm, **(B)** = 2 mm, **(C)** = 100 μm, (**D–H**) = 15 μm, **(I)** = 10 μm, and **(J, K)** = 5 cm.

*Description*: *Saprobic* on a dead branch of *M. domestica* in China. **Sexual morph**: See Fan et al. (2020). **Asexual morph**: *Pycnidial stromata* ostiolate, scattered, immersed in bark, erumpent through the surface of bark, flat, discoid, with multiple locules. *Conceptacle* absent. *Ectostromatic disc* 250–450 µm diam., circular to ovoid, brown, with one to five ostioles per disc. *Ostioles* numerous, 55–105 μm diam., arranged circularly, black, at the same level as the disc. *Locules* numerous, 900–1550 µm diam., irregular, arranged circularly with common walls. *Conidiomata wall* comprising a few layers of cells of *textura angularis*, with innermost layer brown, outer layer brown to dark brown. *Conidiophores* 10–20 × 1.5–2.0 μm, hyaline, unbranched, filamentous, thin-walled. *Conidiogenous cells* 9–16 × 1–2 μm (av. = 13.5 × 1.5 μm, *n* = 20), enteroblastic polyphialidic. *Conidia* 4.5–7 × 1–2 μm (av. = 5.5 × 1.5 μm, *n* = 20), allantoid, aseptate, hyaline, smooth, thin-walled.

*Culture characteristics*: Conidia germinating on PDA, producing germ tubes from both ends within 24 h. Colonies fast growing, reaching up to 6 cm in diam. after 7 days and entirely covering the 9-cm plate after 14 days, centrally white and olivaceous gray at the margin, becoming olivaceous black at the center. Colonies are slightly fluffy, thin with a uniform texture; sterile.

*Material examined*: CHINA, Yunnan Province, Qujing City, 25°47′77″N, 62°34′96″E, on a dead branch of *M. domestica*, 1 October 2021, Guiqing Zhang, Z6 = MHZU 23-0266, living culture: ZHKUCC 23-0980; *Ibid*. Z6-1 = GMB1005, living culture: GMBCC1005.

*Known host and distribution*: Known on *M. pumila* from Hebei, Ningxia, and Gansu provinces, China (Fan et al., 2020); on *Chestnut* from Hebei, China (Jiang et al., 2020); on *M.* sp*ectabilis* from Tibet, China (Li et al., 2024a); and on *M. domestica* from Yunnan Province, China (this study).

*Notes*: Our second cytospora-like taxon morphologically resembles *C. schulzeri* (CFCC 50040 and CFCC 50042), and this was confirmed in the phylogenetic analysis (**Figure 1**, ZHKUCC 23-0980 and GMBCC1005). *Cytospora schulzeri* is a common pathogen that causes apple canker disease in China (Wei, 1979; Zhuang, 2005). *Cytospora schulzeri* was reported as a saprobe on *M. pumila* from Hebei, Ningxia, and Gansu provinces (Fan et al., 2020); as pathogens that caused canker disease in *Castanea mollissima* and branches of *M.* sp*ectabilis* from Hebei Province and Tibet, respectively (Jiang et al., 2020; Li et al., 2024a). In this study, samples were collected from branches of *M. domestica* and identified as a saprobic fungus. Hence, this is the first record of *C. schulzeri* on *M. domestica* from Yunnan Province, southwestern China.

***Allocryptovalsa*** Senwanna, Phookamsak & K.D. Hyde, in Senwanna, Phookamsak, Doilom, Hyde & Cheewangkoon, Mycosphere 8(10): 1839

Index Fungorum Registration Identifier: 553857

Type species: *Allocryptovalsa polyspora* Senwanna, Phookamsak & K.D. Hyde, in Senwanna, Phookamsak, Doilom, Hyde & Cheewangkoon, Mycosphere 8(10): 1840

*Allocryptovalsa* Senwanna, Phookamsak & K.D. Hyde, typified with *A. polyspora* Senwanna, Phookamsak & K.D. Hyde. Zhu et al. (2021) regarded that the members of *Allocryptovalsa* show a cosmopolitan distribution (i.e., in Australia, China, Germany, India, Thailand, and the United States). *Allocryptovalsa* was originally introduced to accommodate two species, i.e., *A. cryptovalsoidea* and *A. rabenhorstii* (basionym: *Valsa rabenhorstii* Nitschke), which was characterized by immersed perithecia, polysporous asci, and allantoid ascospores (Senwanna et al., 2017). Subsequently, Konta et al. (2020) and Hyde et al. (2020) introduced *A. elaeidis* from *Elaeis guineensis* and *A. truncata* isolated from decaying twigs of unidentified plants, respectively. Zhu et al. (2021) introduced two new species within this genus, *A. castaneae* and *A. castaneicola*, which were isolated from *C. mollissima* in China. Hitherto, three additional new species have been reported in *Allocryptovalsa. Allocryptovalsa xishuangbanica* was discovered on dead branches collected from China (Maharachchikumbura et al., 2022); *A. aceris* was isolated on dead twigs of *Acer palmatum* (*Aceraceae*) from China (Senanayake et al., 2023); and *A. aquilariae* was isolated from dead twigs of *Aquilaria sinensis* from China (Chethana et al., 2023). In this study, we collected *A. castaneae* and it is the first host record in China.

***Allocryptovalsa castaneae*** N. Jiang & X.L. Fan, Frontiers in Microbiology 12(no. 646262): 5

Index Fungorum Registration Identifier: 837777

**Figure 7**

**Figure 7:**
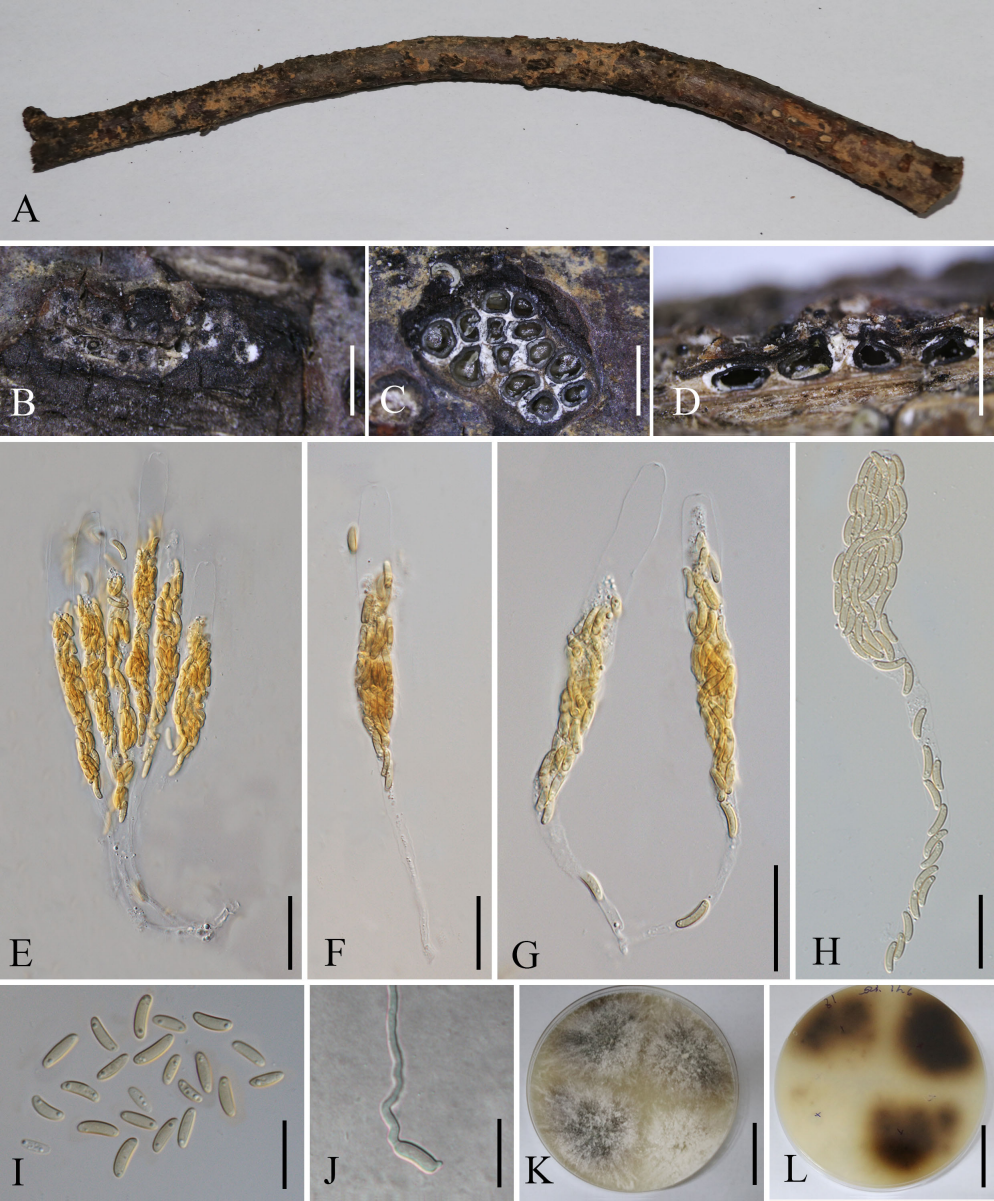
*Allocryptovalsa castaneaea* (MHZU 23-0264). **(A)** *Malus domestica* branch. **(B)** Habit of stromata on a branch. **(C)** Transverse section through stroma. **(D)** Longitudinal section through the stroma. **(E–H)** Asci. **(I)** Ascospores. **(J)** Germinating ascospore. **(K, L)** Colonies on PDA from above and below [**(K)** above, **(L)** below]. Scale bars: **(B)** = 2 mm, **(C)** = 250 μm, **(D)** = 1.2 mm, **(E–H)** = 30 μm, **(I, J)** = 5 μm, and **(K, L)** = 4 cm.

*Description*: Saprobic on branches of *M. domestica* in China. **Sexual morph**: *Stromata* 2.5–3.5 mm diam., scattered to gregarious, immersed in the bark, erumpent through the surface of bark, with 8–13 perithecia arranged irregularly. *Ectostromatic disc* 0.2–0.6 mm diam., circular to oblong, brown, with more than eight ostioles arranged circularly per disc. *Ostioles* numerous, 100–250 µm diam., gregarious, umbilicate, 4-sulcate dark brown to black, at the same level as the disc. *Perithecia* 210–280 µm diam., outer surface coated with yellow, powdery entostroma, black, flask-shaped, perithecial necks erumpent in groups. *Asci* 120–220 × 12–24 µm (av. = 155 × 18 µm, *n* = 10), unitunicate, polysporous, clavate to elongate obovoid, long pedicellate, apically rounded, thin-walled. *Ascospores* 7.5–10.5 × 2.5–3.5 µm (av. = 9 × 3 µm, *n* = 20), aseptate, elongate-allantoid, slightly curved, pale yellowish to pale brown at maturity, smooth, thin-walled. **Asexual morph**: undetermined.

*Culture characteristics*: Colonies are initially white, uniform, becoming dark after 2 weeks.

*Material examined*: CHINA, Yunnan Province, Qujing City, 25°47′77″N, 62°34′96″E, 1 October 2021, on a dead branch of *M. domestica*, Guiqing Zhang, Z7 = MHZU 23-0264, living culture: ZHKUCC 23-0981; *Ibid*. Z1, living culture: ZHKUCC 23-0982.

*Known host and distribution*: Known on *C. mollissima* from Hebei Province and on *Juglans regia* in Yunnan Province, Chuxiong Yi Autonomous Prefecture (Zhu et al., 2021); *M. domestica* in Yunnan province, southwestern China (this study).

*Notes*: *Allocryptovalsa castaneae* is a taxon with a distinctive orange ectostromatic disc, and short pedicellate asci, which was described by Zhu et al. (2021). However, our new collection has black ectostromatic disc and slightly broader asci with a longer pedicel. Nevertheless, based on phylogenetic analysis (**Figure 2**), we confirmed that the new collections (i.e., ZHKUCC 23-0981 and ZHKUCC 23-0982) are *A. castaneae. Allocryptovalsa castaneae* has been known on *C. mollissima* in Hebei and on *J. regia* in Chuxiong Yi Autonomous Prefecture, Yunnan Province, China. Therefore, we report a new host record of *A. castaneae* on *M. domestica* from Qujing City, Yunnan, China.

***Aureobasidium*** Viala & G. Boyer, Rev. gén. Bot. 3: 371

Index Fungorum Registration Identifier: 7297

Type species: *Aureobasidium vitis* Viala & G. Boyer, Rev. gén. Bot. 3: 371

*Aureobasidium* Viala & G. Boyer, typified with *Au. vitis* [current name: *Au. pullulans* (de Bary & Löwenthal) G. Arnaud]. *Aureobasidium* species are often called black yeast because they produce melanin during growth, and species of *Aureobasidium* with a yeast-like morph have a wide range of distribution and diverse life modes, including saprobes, endophytes, and pathogens (Lee et al., 2021; Wang et al., 2022). Recently, based on morphology characters, phylogenetic analysis, and biochemistry, more species of this genus have been introduced. So far, 66 epithets have been recorded in Index Fungorum (2024) (accessed 23 June 2024). *Aureobasidium pullulans* can produce pullulan polysaccharides, which are with the properties of water retention, barrier formation, regeneration, whitening, hydrating, and repairing, and it also serves as an ingredient in cosmetic formulas (Wu et al., 2023). In this study, we collected an *Aureobasidium* species from *M. domestica*. Morpho-molecular analyses confirmed that it is *Au. pullulans.*

***Aureobasidium pullulans*** (de Bary & Löwenthal) G. Arnaud, Annals d’École National d’Agric. de Montpellier, Série 2 16(1-4): 39

Index Fungorum Registration Identifier: 101771

**Figure 8**

**Figure 8:**
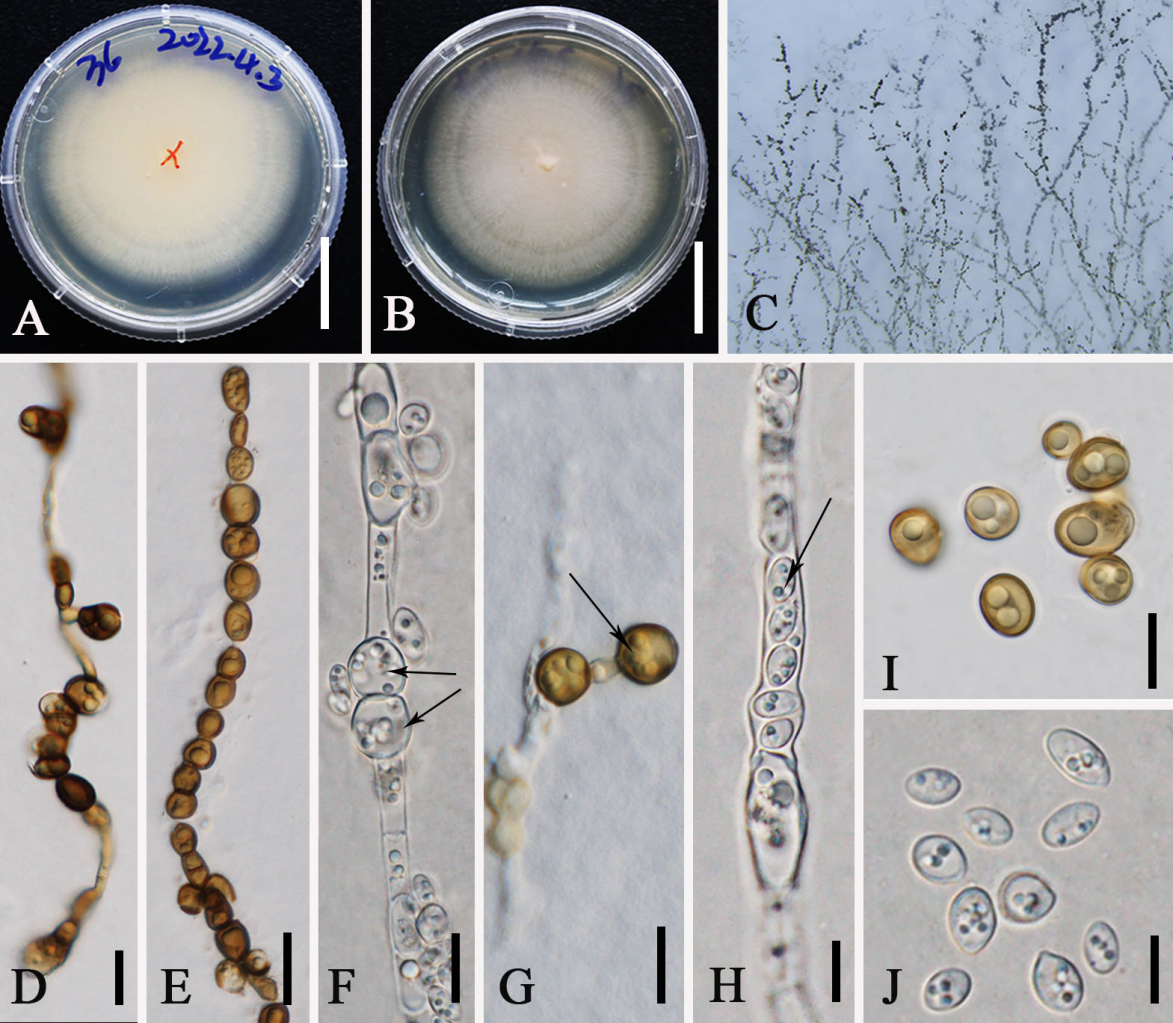
*Aureobasidium pullulans* (ZHKUCC 23-0983). **(A, B)** Colonies on PDA from above and below [**(A)** below, **(B)** above] **(C)** Melanized hyphae. **(D)** Melanized hyphae developing into arthrospores and chlamydospores. **(E)** Melanized hyphae/clamydospores. **(F)** Intercalary clamydospores. **(G)** Terminal clamydospores. **(H)** Endoconidia. **(I)** Chlamydospores. **(J)** Conida. Scale bars: **(A, B)** = 3 cm, **(D, G)** = 15 µm, **(E)** = 20 μm, and **(F–J)** = 10 μm.

*Description*: *Endophytic* in a leaf of *M. domestica* in China. **Sexual morph**: not observed. **Asexual morph on PDA**: Vegetative hyphae hyaline to brown, smooth to slightly roughened, thin-walled, 4–12 µm wide, constricted at septa, disarticulating to dark brown chlamydospores, 7–13 × 8–10 µm, intercalary or terminal. *Conidiogenous cells* undifferentiated, intercalary or terminal, or arising as short lateral branches on hyaline hyphae. *Conidia* 5−10 × 3−5 µm (av. = 8 × 4 μm, *n* = 20), one-celled, ellipsoidal and variable in shape and size, hyaline homogeneous to sectored, yeast-like to filamentous growth, smooth. Secondary conidia smaller. *Endoconidia* 5 × 3 µm, occasionally produced in an intercalary cell and released into a neighboring empty cell.

*Culture characteristics*: Colonies on PDA at 28°C attaining approximately 50 mm diam. after 7 days, appearing smooth and slimy due to abundant sporulation, pinkish, reverse pinkish. Initially white, uniform, becoming pink after 2 weeks. *Hyphae* are hyaline, smooth, thin-walled, with transverse septa.

*Material examined*: CHINA, Yunnan Province, Qujing City, *Malus* plantation, 25°47′77″N, 62°34′96″E, on the leaves of *M. domestica*, 1 October 2021, Guiqing Zhang, Z24, living culture: ZHKUCC 23-0983. *Ibid*. Z36, living culture: GMBCC1006

*Known host and distribution*: Known on *M. domestica* in Poland, Europe (Mirzwa-Mróz and Wińska-Krysiak, 2011); *M. pumila* in Canada (Ginns, 1986); *Hylocereus polyrhizus* and *Hylocereus undatus* (Taylor and Hyde, 2003; Ding et al., 2021); *Pinus thunbergii* (Jayawardena et al., 2018); *Trachycarpus fortune* (Wu et al., 2017); *Vitis* sp (Grabowski, 2007); *Pinus* in China (Ding et al., 2021); and *M. domestica* in Yunnan Province, China (this study).

*Notes*: In this study, *Au. pullulans* was isolated as an endophyte in the leaves of *M. domestica*. Previously, it was reported from the leaves of *M. domestica* which was grown at an experimental orchard in Germany (Rühmann et al., 2013). Moreover, it shows a broad range of geographical distribution from cold to warm climates and wet/humid regions to arid ones (Bozoudi and Tsaltas, 2018). Phylogenetically, our new collections (ZHKUCC 23-0983, GMBCC1006) clustered in the clade that comprises *Au. pullulans*, *Au. proteae*, and *Au. microstictum*. Morphologically, our collections closely resemble *Au. pullulans*, in having yeast-like colonies covered with a slimy mass of spores and same-size conidia [5–10 × 3–5 µm (av. = 8 × 4 µm, *n* = 20)] and possess chlamydospores and endoconidia. However, our collections can be distinguished from *Au. proteae* and *Au. microstictum* by the presence of chlamydospores and endoconidia (Yoshikawa and Yokoyama, 1987; Crous et al., 2011). Thus, we confirmed our new collections (ZHKUCC 23-0983 and GMBCC1006) to belong to *Au. pullulans*, and this is the first report of *Au. pullulans* from Yunnan, southwestern China.

***Hypoxylon*** Bull., Hist. Champ. Fr. (Paris) 1(1): 168

Index Fungorum Registration Identifier: 2456

Type species: *Hypoxylon coccineum* Bull., Hist. Champ. Fr. (Paris) 1(1): 174

Species of *Hypoxylon* are often isolated as saprobes and endophytes of angiospermous plants (Halecker et al., 2020; Ma et al., 2022). The sexual morph of *Hypoxylon* is characterized by hemispherical, cushion-shaped stromata, immersed locules, periphysate ostiolate opening, eight-spored, uniseriate asci with an amyloid apical ring, one-celled ascospores (Rogers, 2018), while asexual morphs are characterized by different branching patterns of conidia, conidiogenous structure with virgariella-like branching patterns on natural substrate and artificial medium (Ju and Rogers, 1996). In this study, we collected a *Hypoxylon* species from apples. Morpho-molecular analyses confirmed that it is a novel taxon of *Hypoxylon*; thus, we introduce *H. malongense.*

***Hypoxylon malongense*** G.Q. Zhang, Wijayaw., & Q.R. Li, **sp. nov.**

Index Fungorum Registration Identifier: IF902663

**Figure 9**

**Figure 9:**
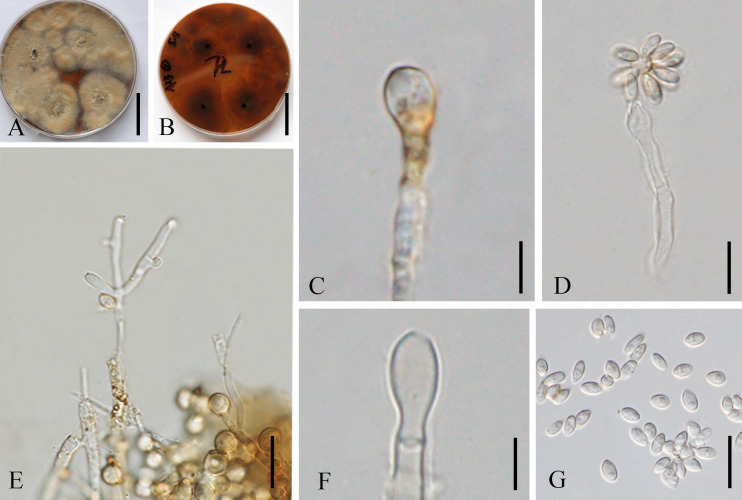
*Hypoxylon malongense* (ZHKUCC 23-0984, ex-type). **(A, B)** Colonies on PDA from above and below [**(A)** below, **(B)** above]. **(C, E, F)** Conidiophores and conidiogenous cells. **(D)** Anamorph from structure with nodulisporium-like branching patterns. **(G)** Conidia. Scale bars: **(A, B)** = 3 cm, **(C)** = 5 µm, **(D, F, G)** = 10 µm, and **(E)** = 15 µm.

*Etymology*: Named after the location “Malong” where the new taxon was first discovered.

*Ex-type*: ZHKUCC 23-0984

*Description*: *Endophytic* in the leaf of *M. domestica* in China. **Sexual morph**: not observed. **Asexual morph on PDA**: Conidiogenous structure on PDA with nodulisporium‐like branching patterns to unbranched. *Conidiophores* simple, septate, thin, sparingly branched or unbranched, straight to slightly curved, hyaline to yellow-brown, smooth walled to sometimes verruculose. *Conidiogenous cells* 9–19 × 2–4 μm (av. = 15 × 3 μm, *n* = 10), enteroblastic, phialidic, terminal or lateral, subcylindrical, straight or slightly curved, hyaline to pale yellow‐brown, smooth to finely verruculose, denticulate and protuberant conidiogenous loci, thickened. *Conidia* 4.1–7 × 2.0–3.0 μm (av. = 5.0 × 2.5 μm, *n* = 20), aseptate, rarely oblong, ellipsoidal to subglobose, apex obtuse, base truncate or bluntly rounded, straight or slightly curved, hyaline, smooth, mostly with minute guttules.

*Culture characteristics*: Colonies on PDA covering a 9-cm Petri dish after 3 weeks at 28°C, under 24 h dark, at first whitish becoming pale brown powdery, with scarce white mycelium that form rays toward the margins; yellowish-brown from below. Sporulating regions scattered over the entire surface of the colony.

*Material examined*: CHINA, Yunnan Province, Qujing City, *M. domestica* plantation, 25°47′77″N, 62°34′96″E, endophytic in the living leaves of *M. domestica*, 1 October 2021, Guiqing Zhang, Z60, ex-type: ZHKUCC 23-0984. *Ibid*. Z72, isotype: GMBCC1007.

*Notes*: The multilocus phylogenetic analyses indicate that our new isolates *H. malongense* [ZHKUCC 23-0984 (ex-type) and GMBCC1007] form an independent lineage sister to *H. hinnuleum* [MUCL 3621 (ex-type), DSM:107932, and DSM:107926] with high statistical support (100% ML, 1.00 PP) (**Figure 4**). Morphologically, *H. malongense* can be distinguished from *H. hinnuleum* by its distinct differences in conidiogenous structure and conidia (**Table 3**). Our isolation is characterized by having nodulisporium-like branching patterns or unbranched conidiogenous structure, with ellipsoidal to subglobose conidia. However, *H. hinnuleum* is characterized by virgariella-like, nodulisporium-like, and periconiella-like branching patterns, with ellipsoid conidia. Therefore, more morphological differences between the new taxon and *H. hinnuleum* are listed in **Table 3**. Based on both morphology and phylogeny, we established this species as a novel taxon within *Hypoxylon*.

**Table 3:** Diagnostic characteristics of *Hypoxylon* species.

Morphological and colony characters	Species name and references	
	*H. hinnuleum* (Sir et al., 2019)	*H. malongense* (This study)
**Conidiogenous structure**	With virgariella-like, nodulisporium-like, and periconiella-like branching patterns	With nodulisporium-like branching patterns to unbranched
**Conidiophores**	Hyaline, smooth to slightly roughened	Hyaline to yellow-brown, septate, smooth-walled to sometimes verruculose, sparingly branched or unbranched
**Conidiogenous cells**	Hyaline, 9.1–20.8 × 1.7–2.4 μm	Hyaline to pale yellow-brown, 9–19 × 2–4 μm, phialidic
**Conidia**	Ellipsoid, hyaline to pale brown, smooth to rough, 3.6–5.1 × 2.1–3.1 μm	Rarely oblong, ellipsoidal to subglobose, apex obtuse, base truncate or bluntly rounded, 4.1–7 × 2.0–3.0 μm
**Culture characteristics**	Colonies on OA covering a 6-cm Petri dish after 3 weeks, at first whitish becoming apricot powdery with scarce white mycelium that forms rays toward the margins; reverse, orange, sienna	Colonies on PDA covering a 9-cm Petri dish after 3 weeks at 28°C, under 24 h dark, at first whitish becoming pale brown powdery, with scarce white mycelium that forms rays toward the margins; yellowish-brown from below

### 3.3 Discussion

#### 3.3.1 Rich and underexplored fungal diversity in Yunnan

Yunnan Province in China is rich in fungal diversity, and annually, a significant number of species are introduced (e.g., Feng and Yang, 2018; Wijayawardene et al., 2021a, b; He and Zhao, 2022; Hongsanan et al., 2023; Du et al., 2023; Tian et al., 2024). These novel species have been reported from well-studied hosts (e.g., 1. from coffee *fide* Lu et al., 2022; 2. from bamboo *fide* Dai et al., 2022; Han et al., 2024a, b; 3. from Pará Rubber *fide* Xu et al., 2024; 4. from *Macadamia fide* Zhang et al., 2024a), well-studied microhabitats (e.g., freshwater fungi *fide* Shen et al., 2022; Li et al., 2024b), and unusual or understudied microhabitats (e.g., fungi from bats *fide* Liu et al., 2023; fungi from dead American bullfrog larvae *fide* Yang et al., 2023a). Furthermore, some species have been introduced from well-studied and complex genera, such as *Colletotrichum*, and confirmed the unexplored and rich fungal diversity in the region (e.g., *Colletotrichum gardeniae* Q. Zhang et al. *fide* Zhang et al., 2023).

Extensive exploration of fungal diversity in Qujing City, Yunnan Province has been carried out since 2019 and more than 16 novel fungal species have been introduced from different hosts in different studies (Yasanthika et al., 2020; Doilom et al., 2021; Monkai et al., 2021; Dissanayake et al., 2022; Wijayawardene et al., 2022b; Yang et al., 2023b, 2024; Wang et al., 2024b; Zhang et al., 2024b; Zhou et al., 2024). The present study is a continuation of a long-term study of discovering fungal diversity in an understudied geographical region, Qujing City. *Malus* species are an important fruit crop that is widely cultivated in Qujing City and its adjacent villages (e.g., Tongquan Town, Maguohe Town, and Wangjiashizhuang Town). There are several varieties of *Malus* species commonly cultivated in this region. Even though *Malus* species are extensively cultivated, there are no proper studies carried out to understand the mycobiota inhabiting its phyllosphere and mycosphere. This study has been carried out to fulfil this requirement. We have not observed any significant diseases; thus, we focused on identifying common saprobic and endophytic taxa associated with *Malus* species. In total, we have isolated 60 species from different localities of Qujing, and most taxa belong to *Alternaria* sp. and *Fusarium* sp.

In the present study, two novel species (*C. qujingensis* and *H. malongense*) and three new records (*C. schulzeri*, *A. castaneae*, and *Au. pullulans*) have been compiled. Based on detailed morphological studies, complemented by phylogenetic analyses based on ITS, *tef*1-α, *rpb*2, *tub*, and *act* sequence data (**Figure 1**), *C. qujingensis* is introduced as a novel saprobe taxon, while collections (ZHKUCC 23-0980 and GMBCC1005) revealed to be hitherto known species, namely, *C. schulzeri*, which was first reported from southwestern China. However, *H. malongense* was isolated as novel endophytic fungi on *M. domestica* from Qujing, Yunnan, based on morphological descriptions coupled with phylogenetic analyses (ITS, LSU, *rpb*2, and *tub* loci regions). Furthermore, Zhu et al. (2021) reported *A. castaneae* on *C. mollissima* and *J. regia* from Hebei and Yunnan provinces, respectively. Herein, we report our collections (ZHKUCC 23-0981 and ZHKUCC 23-0982) as a new host record of *A. castaneae* isolated from *M. domestica*. Moreover, the ITS and LSU regions are used to construct the phylogenetic tree of *Aureobasidium*, and the placements in **Figure 3** in our study agree with Humphries et al. (2017) and Wu et al. (2023), who performed their analysis based on LSU and ITS gene regions. The morphological characteristics of each related species in the phylogenetic tree are compared in our study. Thus, we regard our collections (ZHKUCC 23-0983 and GMBCC1006) as the first report of *Au. pullulans* on *M. domestica* from Yunnan, China. Both the geographical and host distribution of fungus provide new insights into its ecological preferences and contribute to our knowledge of its regional distribution (Li et al., 2024b).

#### 3.3.2 Species that can switch life modes; insights from this study

Fungi play key roles in ecosystems as saprobes, endophytes, and pathogens, but the role of an individual species in nature is still unknown (Schmit and Mueller, 2007). Recently, more research focused on the properties of endophytic fungi. For example, the secondary metabolites produced by endophytes can be employed in biotechnology such as in the pharmaceutical industry (Strobel and Daisy, 2003; Mapook et al., 2022). Mattoo and Nonzom (2021) mentioned that the endophyte could switch to be a pathogen lifestyle when it became more widespread upon the host getting old (e.g., *Colletotrichum tropicale fide* Rojas et al., 2010). For example, *Pestalotiopsis palmarum*, *Ceratocystis paradoxa*, and *Ganoderma lucidum* have been reported as endophytes, pathogens, or saprobes on coconut, which suggested that those fungi may switch their lifestyles from endophytes to pathogens or saprobes (Bhunjun et al., 2022; Tian et al., 2024). Furthermore, Knapp et al. (2018) claimed that a saprobic ancestral lifestyle of endophytes would be the reason for its ability to produce enzymes. Bhunjun et al. (2024) argued that the endophytic lifestyle is the ancestor of fungi, implying that some endophytes are host-specific, while others are connected to a wide range of hosts. A better understanding of fungi lifestyle switching could help us fill the gaps on fungi diversity and secondary metabolite diversity produced by fungi. Glauser et al. (2009) predicted that the production of secondary metabolite can be triggered by the presence of other fungi and pathogens.

In the present study, we isolated two *Cytospora* species, and both of them are isolated as saprobic taxa on *M. domestica*. More than 100 species of *Cytospora* are causal agents (or associated with) of the stem canker and dieback of woody and coniferous plants (e.g., *Populus* and *Salix*) (Fan et al., 2020). Initially, *C. schulzeri* was reported as a pathogen that can cause black spots on the infected apple trees in China (Wei, 1979; Zhuang, 2005). Recently, it was reported as a saprobe on *M. pumila* and a pathogen on *C. mollissima* and *M. spectabilis* in China (Fan et al., 2020; Jiang et al., 2020; Li et al., 2024a). However, we have not observed any disease symptoms that are similar to symptoms caused by *C. schulzeri* on *Malus* spp. in our study areas. Thus, we conclude that *C. schulzeri* is only a saprobic taxon in this region. More collections are needed to confirm whether *C. qujingensis* and *C. schulzeri* can exist as endophytes. Furthermore, pathogenicity tests need to be carried out to check whether both species are latent pathogens of *M. domestica*.

Species of *Diatrypaceae* were less reported as pathogens (Trouillas et al., 2011) or endophytes in petioles and woody tissue (Carroll, 1986; de Errasti et al., 2010), but most of them were predominantly saprobes inhibiting the wood and bark of various angiosperms. Hitherto, *Allocryptovalsa* species have not been reported as pathogens. Nevertheless, we conclude that further collection of different hosts in this region would provide insightful data to conclude the host switching and life mode changes of the taxon.

*Aureobasidium pullulans* was initially found as a saprobic yeast-like taxon but subsequently reported as an endophyte on the flesh of sweet cherries (Schena et al., 2003) and a pathogen isolated from the environment of a patient’s bone marrow (Liu et al., 2020). Ginns (1986) isolated this species on *M. pumila* from Canada, and later, Mirzwa-Mróz and Wińska-Krysiak (2011) recorded *Au. pullulans* on *M. domestica* from Europe. Previously, Ding et al. (2021) reported *Au. pullulans* from *P. thunbergii* (which causes brown spot needle blight) in China. In our study, we report *Au. pullulans* as an endophyte on *M. domestica* from China for the first time. Furthermore, many yeast species, including the yeast-like species, *Au. pullulans*, have been reported as effective antagonists against postharvest diseases in fruit. *Aureobasidium pullulans* is an effective biocontrol agent against postharvest diseases in various fruits, including apples (Castoria et al., 2001).

*Hypoxylon malongense* was isolated as an endophyte, from *M. domestica* in Qujing in the present study. *Hypoxylon* species are generally regarded as saprobe, but some of them are known to have an endophytic phase in their life cycle (viz., *H. rubiginosum*, *H. guilanense*) (Halecker et al., 2020). For example, *H. monticulosum* and *H. submonticulosum* have been isolated as endophytes from the stems of *Litsea akoensis* var. *chitouchiaoensis* (*Lauraceae*) and raspberry (*Rubus idaeus*), respectively (Burgess et al., 2017; Cheng et al., 2020). Furthermore, *H. fuscum*, *H. truncatum*, and *H. investiens* have been reported to exist with an endophytic lifestyle and produce abundant secondary metabolites (Gu et al., 2007; Basnet et al., 2019; Yuan et al., 2019). Some species in this genus are found as pathogens on woody plants. For example, *H. macrocarpum* was reported as the causal agent of wood rot (Hu and Wright, 2022). More studies indicated that *Hypoxylon* can depend on a wide range of hosts (Ma et al., 2022; Zhu et al., 2023). However, whether there is lifestyle switching, more samples are needed to demonstrate the mechanisms of switching between and within species. Anyway, illustrations and morphological descriptions of the asexual morph of this genus in the future are also needed. More research is still needed to figure out the species of our collections whether they contain secondary metabolites to further determine their classification placements. However, we believe it is essential to document and make an inventory of the distribution of species to understand the biogeographical and evolutionary patterns.

#### 3.3.3 Necessity of inventorying and continuous updating of *Malus*-associated fungi against the geographical distribution

Nevertheless, the fungal diversity and impact of fungal pathogens on the agricultural and forest industries are poorly studied in China. As Shen et al. (2018) summarized, fungal diversity will be affected by plant species, environmental conditions, sampling time, and isolation and extraction methods.

Shen et al. (2018) indicated the remarkable fungal diversity on the apple surfaces. Dai et al. (2021) listed all pathogenic fungi on apples (containing apple root, shoot, leaf, flower, and fruit). There exist 65 types of apple disease in China, and 46 of them are caused by 149 different kinds of pathogenic fungal species. A search involving the keywords “*Malus domestica*” and “China” retrieved 47 fungi species present in the USDA Fungal Databases (accessed on 20 September 2024). Furthermore, extra retrieval involving the keywords “*Malus domestica*” and “China” retrieved 19 titles of research articles that had been published since 2018 in the Scopus Database (accessed on 20 December 2024). We scanned more than 100 available articles about fungi species (including saprobes, pathogens, and endophytes) associated with *M. domestica* reported in China in Chinese and English from 2000 to present. Hitherto known fungi species isolated on *M. domestica* from China, including saprobic, pathogenic, and endophytic fungi, was presented in a checklist (**Supplementary Table S1**), which will expand our knowledge of fungi associated with *M. domestica* and will prove timely and informative to a researcher’s future work.

Some culturable taxa like *Alternaria*, *Aspergillus*, *Cladosporium*, and *Penicillium* have been identified by previous studies (Magnani et al., 2007; Tadych et al., 2012). Granado et al. (2008) pointed out that the molecular approach, involving ribosomal DNA sequencing, to identify yeasts and sterile fungi, allows to spot unculturable fungi on apple fruits in the future. Bulgari et al. (2012) reported some uncultured species on *Malus*, viz., *Bradyrhizobium*, *Bradyrhizobium*, and *Legionella.* Our current study showed that *Malus* species are rich in culturable fungal diversity. However, we have not regarded the unculturable fungal diversity in this study. Hence, we conclude that more studies need to be carried out to collect more data to recognize the distribution of *Malus* taxa, and for precise identification based on the morpho-molecular analyses, barcoding current pathogens and potential pathogens would also be essential. Furthermore, more samples are required to determine whether fungi exist as a saprobe or endophyte and have the potential to be pathogens as well. In the future, studies based on omics approaches would be helpful in discovering novel taxa including unculturable taxa (Wijayawardene et al., 2023).

## Data Availability

The datasets presented in this study can be found in online repositories. The names of the repository/repositories and accession number(s) can be found in the article/**Supplementary Material**.

## Supplementary material

Supplementary Table 1 Checklist of fungi associated with *Malus domestica*/*Malus pumila* in China.

Supplementary Table 3 The names, isolate numbers, and corresponding GenBank accession numbers of the taxa used in **Figure 1**.

Supplementary Table 4 The names, isolate numbers, and corresponding GenBank accession numbers of the taxa used in **Figure 2**.

Supplementary Table 5 The names, isolate numbers, and corresponding GenBank accession numbers of the taxa used in **Figure 3**.

Supplementary Table 6 The names, isolate numbers, and corresponding GenBank accession numbers of the taxa used in **Figure 4**.
