# Supplementary material for: Fungi from Malus in Qujing, China: two new species, three new records, and insights into potential host jumping and lifestyle switching
Source: Front Cell Infect Microbiol. 2025 Mar 11;15:1517908. doi: 10.3389/fcimb.2025.1517908 (PMC11933099; doi:10.3389/fcimb.2025.1517908)
Supplement: Supplementary Table 1 — Checklist of fungi associated with Malus domestica/Malus pumila in China. [file Table1.docx]

Supplementary Material

# Supplementary Figures and Tables

**TABLE S1:** Checklist of fungi associated with *Malus domestica*/*Malus pumila* in China

| Species | Host | Origin |
| --- | --- | --- |
| *Acremonium sclerotigenum* | *Malus domestica* | Shandong |
| *A. sclerotigenum* | *Malus domestica* | Shaanxi |
| *Alternaria alternata* | *Malus pumila* | Hebei |
| *A. alternata* | *Malus pumila* | / |
| *A. alternata* | *Malus pumila* | / |
| *A. mali* | *Malus pumila* | / |
| *A. mali* | *Malus pumila* | Hebei |
| *A. mali* | *Malus pumila* | / |
| *A. mali* | *Malus pumila* | / |
| *A. malicola* | *Malus domestica* | Shaanxi |
| *A. pomicola* | *Malus pumila* | Shandong |
| *A. pomicola* | *Malus pumila* | Beijing |
| *A. pomicola* | *Malus pumila* | / |
| *A. pomicola* | *Malus pumila* | Hebei |
| *A.* sp. | *Malus domestica* | Shaanxi |
| *A. tenuissima* | *Malus pumila* | / |
| *A. tenuissima* | *Malus pumila* | / |
| *Allocryptovalsa castaneae* | *Malus domestica* | Yunnan |
| *Aplosporella mali* | *Malus pumila* | / |
| *Ascochyta mali* | *Malus pumila* | Hebei |
| *A. malvicola* | *Malus sylvestris* | Hebei |
| *A. piricola* | *Malus pumila* | / |
| *Aspergillus awamori* | *Malus pumila* | / |
| *A. foetidus* | *Malus pumila* | / |
| *A. fumigatus* | *Malus pumila* | / |
| *A. niger* | *Malus pumila* | / |
| *Aureobasidium pullulans* | *Malus domestica* | Yunnan |
| *Auricularia mesenterica* | *Malus* sp. | / |
| *Biscogniauxia mandshurica* | *Malus* sp. | Heilongjiang |
| *Botryosphaeria dothidea* | *Malus domestica* | Beijing/Henan/Liaoning/Shandong/Shanxi/Shaanxi/Yunnan |
| *B. dothidea* | *Malus domestica* | Shaanxi |
| *B.* *dothidea* | *Malus domestica* | Shaanxi |
| *B. kuwatsukai* | *Malus domestica* | Shaanxi |
| *B. ribis* | *Malus pumila* | / |
| *B. rosaceae* | *Malus* sp. | Fujian |
| *B. rosaceae* | *Malus* sp. | Shandong/Henan/Beijing |
| *B. sinensia* | *Malus pumila* | Beijing |
| *Botrytis cinerea* | *Malus pumila* | / |
| *Cercospora hyalina* | *Malus pumila* | / |
| *C. hyalina* | *Malus pumila* | / |
| *C. malvarum* | *Malus sylvestris* | / |
| *Cladosporium cladosporioides* | *Malus pumila* | / |
| *C.* sp. | *Malus domestica* | Shaanxi |
| *C. sphaerospermum* | *Malus pumila* | / |
| *Colletotrichum acutatum* | *Malus domestica* | Shaanxi/Henan |
| *C. aenigma* | *Malus domestica* | / |
| *C. asianum* | *Malus domestica* | Suizhong/Liaoning |
| *C. fructicola* | *Malus domestica* | Henan |
| *C. fructicola* | *Malus domestica* | Xian |
| *C. fructicola* | *Malus domestica* | / |
| *C. rhombiforme* | *Malus domestica* | Sichuan |
| *Coniothecium chomatosporum* | *Malus pumila* | / |
| *Coniothyrium pyrinum* | *Malus pumila* | / |
| *C. tirolense* | *Malus pumila* | / |
| *C. tirolense* | *Malus pumila* | / |
| *Coriolus versicolor* | *Malus pumila* | / |
| *Corticium centrifugum* | *Malus pumila* | / |
| *C. salmonicolor* | *Malus pumila* | / |
| *C. foliicola* | *Malus pumila* | / |
| *Cylindrocarpon mali* | *Malus pumila* | / |
| *C. mali* | *Malus pumila* | / |
| *C. leucostoma* | *Malus pumila* | / |
| *Cytospora leucostoma* | *Malus sieversii* | / |
| *C. leucostoma* | *Malus domestica* | Xian |
| *C. mali* | *Malus pumila* | / |
| *C. mali* | *Malus sieversii* | / |
| *C. mali* | *Malus sieversii* | Xinjiang Uygur Autonomous Region |
| *C. parasitica* | *Malus* sp. | Xinjiang Uygur Autonomous Region |
| *C. parasitica* | *Malus sieversii* | Xinjiang Uygur Autonomous Region |
| *C. parasitica* | *Malus sieversii* | Xinjiang Uygur Autonomous Region |
| *C. parasitica* | *Malus pumila* | Xinjiang Uygur Autonomous Region |
| 1. *qujingensis* | *Malus domestica* | Yunnan |
| *C. sacculus* | *Malus pumila* | Shaanxi/Gansu/Ningxia |
| *C. schulzeri* | *Malus domestica* | Yunnan/Xinjiang Uygur Autonomous Region |
| *C.* sp. | *Malus pumila* | / |
| *C.* sp. | *Malus sieversii* | / |
| *Daedalea biennis* | *Malus* sp. | / |
| *D. biennis* | *Malus* sp. | / |
| *Diaporthe eres* | *Malus pumila* | Shandong |
| *D. nobilis* | *Malus pumila* | Shandong |
| *D. pomigena* | *Malus pumila* | / |
| *Diplodia mutila* | *Malus pumila* | / |
| *Dissoconium mali* | *Malus pumila* | Shanxi |
| *D. proteae* | *Malus domestica* | Shaanxi |
| *Endomycopsis mali* | *Malus pumila* | / |
| *Fomes fomentarius* | *Malus pumila* | / |
| *F. fomentarius* | *Malus* sp. | / |
| *Fusarium avenaceum* | *Malus pumila* | / |
| *F. avenaceum* | *Malus sieversii* | Xinjiang Uighur Autonomous Region |
| *F. camptoceras* | *Malus pumila* | / |
| *F. moniliforme* | *Malus pumila* | / |
| *F. proliferatum* | *Malus sieversii* | Xinjiang Uighur Autonomous Region |
| *F. proliferatum* | *Malus pumila* | Shandong |
| *F. solani* | *Malus pumila* |  |
| *F. solani* | *Malus sieversii* | Xinjiang Uighur Autonomous Region |
| *F.* sp. | *Malus domestica* | Shaanxi |
| *F.* sp. | *Malus pumila* | / |
| *F.* sp. | *Malus sieversii* | Xinjiang Uighur Autonomous Region |
| *F. sporotrichioides* | *Malus sieversii* | Xinjiang Uighur Autonomous Region |
| *F. tricinctum* | *Malus sieversii* | Xinjiang Uighur Autonomous Region |
| *Fusicladium dendriticum* | *Malus pumila* | / |
| *F. dendriticum* | *Malus pumila* | / |
| *F. pomi* | *Malus domestica* | / |
| *F. pomi* | *Malus sylvestris* | / |
| *F. pomi* | *Malus* sp. | / |
| *Fusicoccum aesculi* | *Malus pumila* | Qihai |
| *Gloeodes pomigena* | *Malus pumila* | / |
| *Gloeosporium album* | *Malus pumila* | / |
| *G. rufomaculans* | *Malus pumila* | / |
| *Glomerella acutata* | *Malus* sp. | Hunan |
| *G. cingulata* | *Malus domestica* | / |
| *G. cingulata* | *Malus pumila* | / |
| *Guignardia pyricola* | *Malus* sp. | / |
| *Gymnosporangium globosum* | *Malus* sp. | / |
| *G. yamadae* | *Malus* sp. | / |
| *G. yamadae* | *Malus* sp. | / |
| *G. yamadae* | *Malus pumila* | / |
| *G. yamadae* | *Malus pumila* | Shaanxi |
| *G. yamadae* | *Malus pumila* | / |
| *G. yamadae* | *Malus pumila* | Shaanxi |
| *G. yamadae* | *Malus pumila* | / |
| *G. yamadae* | *Malus pumila* | / |
| *Haplosporella ailanthi* | *Malus pumila* | / |
| *Helicobasidium mompa* | *Malus pumila* | / |
| *Hendersonia mali* | *Malus pumila* | / |
| *Houjia yanglingensis* | *Malus* sp. | Zhejiang/Henan |
| *Hypoxylon malongense* | *Malus domestica* | Yunnan |
| *Inonotus hispidus* | *Malus* sp. | / |
| *Lasiodiplodia pseudotheobromae* | *Malus pumila* | Shandong |
| *Lenzites tricolor var. daedalea* | Malus sp. | / |
| *Leptosphaeria mandshurica* | *Malus pumila* | / |
| *L. pomi* | *Malus pumila* | / |
| *L. pomi* | *Malus pumila* |  |
| *Leucostoma persoonii* | *Malus domestica* | / |
| *M. kawatsukai* | *Malus pumila* | / |
| *M. kawatsukai* | *Malus pumila* | / |
| *Marssonina coronaria* | *Malus pumila* | / |
| *M. mali* | *Malus pumila* | / |
| *M. mali* | *Malus pumila* | / |
| *M. mali* | *Malus pumila var. tomentosa* | / |
| *M. mali* | *Malus* sp. | / |
| *Monilia polystroma* | *Malus domestica* | Shandong |
| *M. yunnanensis* | *Malus domestica* | Yunnan |
| *M. yunnanensis* | *Malus domestica* | Shandong |
| *Monilinia fructicola* | *Malus domestica* | Shandong |
| *M. fructicola* | *Malus pumila* | / |
| *M. fructigena* | *Malus pumila* | / |
| *M. mali* | *Malus* sp. | / |
| *Monochaetia unicornis* | *Malus pumila* | / |
| *Monodictys melanopa* | *Malus pumila* | / |
| *Mycosphaerella pomacearum* | *Malus pumila* | / |
| *Oidium* sp. | *Malus pumila* | / |
| 1. sp*.* | *Malus sieversii* | / |
| *O.* sp. | *Malus* sp. | / |
| *Paecilomyces niveus* | *Malus pumila* | Beijing |
| *Peltaster fructicola* | *Malus domestica* | / |
| *Penicillium chrysogenum* | *Malus domestica* | / |
| *P. cyclopium* | *Malus pumila* | / |
| *P. digitatum* | *Malus domestica* | Shaanxi |
| *P. expansum* | *Malus pumila* | / |
| *P. expansum* | *Malus pumila* | / |
| *P. expansum* | *Malus domestica* | / |
| *P. frequentans* | *Malus pumila* | / |
| *P. islandicum* | *Malus pumila* | / |
| *P. paneum* | *Malus domestica* | / |
| *P. viridicatum* | *Malus domestica* | / |
| *Pestalotia breviseta* | *Malus pumila* | / |
| *P. malicola* | *Malus pumila* | / |
| *Pestalotiopsis malicola* | *Malus pumila* | / |
| *Phellinus pomaceus* | *Malus* sp. | / |
| *Phoma glomerata* | *Malus domestica* | Shaanxi |
| *P. pomi* | *Malus pumila* | / |
| *P.* sp. | *Malus domestica* | Shaanxi |
| *Phomopsis fukushii* | *Malus pumila* | Shandong |
| *P. truncicola* | *Malus pumila* | / |
| *P. mali* | *Malus pumila* | / |
| *P. mali* | *Malus pumila* | / |
| *P. pirina* | *Malus pumila* | / |
| *P. pirina* | *Malus pumila* | / |
| *Phyllosticta pirina* | *Malus pumila* | / |
| *P. solitaria* | *Malus pumila* | / |
| *P. solitaria* | *Malus pumila* | / |
| *P. tumanensis* | *Malus pumila* | / |
| *Physalospora obtusa* | *Malus pumila* | / |
| *Phytophthora cactorum* | *Malus pumila* | / |
| *P. cactorum* | *Malus pumila* | / |
| *Phytophthora nicotianae* | *Malus pumila* | / |
| *Phytophthora plurivora* | *Malus sieversii* | Xinjiang Uygur Autonomous Region |
| *Pithomyces chartarum* | *Malus pumila* | / |
| *Podosphaera leucotricha* | *Malus* sp. | / |
| *P. leucotricha* | *Malus* sp. | / |
| *P. leucotricha* | *Malus* sp. | / |
| *P. leucotricha* | *Malus pumila* | / |
| *P. leucotricha* | *Malus pumila* | / |
| *P. leucotricha* | *Malus pumila* | / |
| *P. leucotricha* | *Malus sieversii* |  |
| *P. leucotricha* | *Malus sieversii* | / |
| *P. leucotricha* | *Malus sieversii* | / |
| *P. leucotricha* | *Malus pumila* | / |
| *P. leucotricha* | *Malus niedzwetzkyana* | / |
| *Polystictus versicolor* | *Malus* sp. | / |
| *Pseudocercospora mali* | *Malus* sp. | / |
| *P. mali* | *Malus* sp. | Leaf spot. |
| *P. mali* | *Malus pumila* | / |
| *Pseudoveronaea ellipsoidea* | *Malus domestica* | Shaanxi |
| *Pyropolyporus fomentarius* | Malus sp. | / |
| *Ramichloridium apiculatum* | *Malus domestica* | Jiangsu |
| *R. luteum* | *Malus domestica* | Shandong |
| *R. luteum* | *Malus domestica* | Beijing |
| *Ramichloridium mali* | *Malus pumila* | Shaanxi |
| *Rhizoctonia solani* | *Malus pumila* | / |
| *Rhizopus oryzae* | *Malus pumila* | Beijing |
| *Rosellinia necatrix* | *Malus pumila* | / |
| *Sarocladium liquanensis* | *Malus domestica* | Shanxi |
| *S. mali* | *Malus domestica* | Shanxi |
| *Scleroramularia henaniensis* | *Malus* sp. | Henan |
| *S. shaanxiensis* | *Malus* sp. | Shaanxi |
| *Sclerotinia kenjiana* | *Malus pumila* | / |
| *S. mali* | *Malus pumila* | / |
| *Sclerotium delphinii* | *Malus domestica* | / |
| *S. rolfsii* | *Malus pumila* | / |
| *Sclerotium rolfsii var. delphinii* | *Malus domestica* | / |
| *Sphaeropsis malorum* | *Malus pumila* | / |
| *Stagonospora prominula* | *Malus pumila* | / |
| *Stemphylium amaranthi* | *Malus sieversii* | Sinkiang |
| *S. mali* | *Malus sieversii* | Yili, Sinkiang |
| *S. microsporum* | *Malus sieversii* | Sinkiang |
| *S. vesicarium* | *Malus sieversii* | Sinkiang |
| *Strelitziana mali* | *Malus domestica* | Yangling and Qianxian in Shaanxi |
| *Trametes gallica* | *Malus* sp. |  |
| *T. gallica var. trogii* | *Malus pumila* | */* |
| *T. malicola* | *Malus* sp. | / |
| *T. versicolor* | *Malus* sp. | / |
| *Trichothecium roseum* | *Malus pumila* |  |
| *T. roseum* | *Malus pumila* | / |
| *Trimmatostroma sp.* | *Malus pumila* | / |
| *Valsa ceratosperma* | *Malus pumila* | Shaanxi |
| *V. mali* | *Malus pumila* | / |
| *V. mali var. mali* | *Malus pumila* | Shaanxi |
| *V. mali var. mali* | *Malus domestica* | Shaanxi, Hebei, Xinxiang, Henan, Lanzhou, Gansu, Ningxia, Shandong |
| *V. mali var. pyri* | *Malus pumila* | Shaanxi |
| *V. mali var. pyri* | *Malus pumila* | Yangling, Shaanxi |
| *V. mali var. pyri* | *Malus domestica* | Yangling, Shaanxi |
| *Valsa malicola* | *Malus pumila* | Shaanxi |
| *V. malicola* | *Malus domestica* | Qishan, Shaanxi |
| *V. malicola* | *Malus domestica* | Qishan,Shaanxi |
| *Valsa persoonii* | *Malus domestica* | Yangling, Shaanxi |
| *Venturia inaequalis* | *Malus pumila* | / |
| *V. inaequalis* | *Malus pumila* | / |
| *Wallemia sebi* | *Malus* sp. | Shaanxi |
| *X. hispidus* | *Malus pumila* | / |
| *Zygophiala cryptogama* | *Malus domestica* | Shaanxi |
| *Z. cryptogama* | *Malus* sp. | Hunan |
| *Z. cylindrica* | *Malus domestica* | Shaanxi |
| *Z. cylindrica* | *Malus domestica* | Hunan |
| *Z. emperorae* | *Malus domestica* | Hunan |
| *Z. qianensis* | *Malus* sp. | Shaanxi |
| *Z. qianensis* | *Malus* sp. | Hunan |
| *Z. qianensis* | *Malus domestica* | Hunan |
| *Z. wisconsinensis* | *Malus domestica* | Hunan |

**TABLE S2:** The names, isolate numbers, and corresponding GenBank accession numbers of the taxa used in figure 1. The taxa produced in this study are indicated in bold, and the type strains are indicated in bold with “T”. “/”: no data available in GenBank.

| **Species name** | **Isolate No.** | **GenBank accession No.** | | | | |
| --- | --- | --- | --- | --- | --- | --- |
|  |  | **ITS** | ***rpb2*** | ***tef*1-α** | ***tub2*** | ***act*** |
| *Cytospora ailanthicola* | CFCC 89970 | MH933618 | MH933592 | MH933494 | MH933565 | MH933526 |
| *C. ailanthicola* | CFCC 59446 | OR826163 | OR832018 | OR832040 | OR832062 | OR831996 |
| *C. albodisca* | CFCC 53161 | MW418406 | MW422909 | MW422921 | MW422933 | MW422899 |
| *C. albodisca* | CFCC 54373 | MW418407 | MW422910 | MW422922 | MW422934 | MW422900 |
| *C. albodisca* | CFCC 59467 | OR826179 | OR832034 | OR832056 | OR832076 | OR832012 |
| *C. albodisca* | CFCC 59537 | OR826180 | OR832035 | OR832057 | OR832077 | OR832013 |
| *C. alba* | CFCC 55462^T^ | MZ702593 | OK303516 | OK303577 | OK303644 | OK303457 |
| *C. alba* | CFCC 55463^T^ | MZ702596 | OK303517 | OK303578 | OK303645 | OK303458 |
| *C. ampulliformis* | MFLUCC 16-0583^T^ | KY417726 | KY417794 | / | / | KY417692 |
| *C. ampulliformis* | MFLUCC 16-0629 | KY417727 | KY417795 | / | / | KY417693 |
| *C. amydgali* | CBS 144233^T^ | MG971853 | / | MG971659 | MG971718 | MG972002 |
| *C. atrocirrhata* | CFCC 89615 | KR045618 | KU710946 | KP310858 | KR045659 | KF498673 |
| *C. atrocirrhata* | CFCC 89616 | KR045619 | KU710947 | KP310859 | KR045660 | KF498674 |
| *C. atrocirrhata* | CXY 1401 | JX534242 | / | / | KM034904 | / |
| *C. atrocirrhata* | CXY 1402 | JX534243 | / | / | KM034903 | / |
| *C. avicennae* | IRAN 4199C^T^ | MW295650 | MW824358 | MW394145 | / | MZ014511 |
| *C. avicennae* | IRAN 4625C | OM368648 | / | OM372510 | / | / |
| *C. azerbaijanica* | IRAN 4201C^T^ | MW295526 | MW824360 | MW394147 | / | MZ014513 |
| *C. azerbaijanica* | IRAN 4627C | OM368650 | / | OM372512 | / | / |
| *C. beilinensis* | CFCC 50493^T^ | MH933619 | / | MH933495 | MH933561 | MH933527 |
| *C. beilinensis* | CFCC 50494 | MH933620 | / | MH933496 | MH933562 | MH933528 |
| *C. berberidis* | CFCC 89927^T^ | KR045620 | KU710948 | KU710913 | KR045661 | KU710990 |
| *C. berberidis* | CFCC 89933 | KR045621 | KU710949 | KU710914 | KR045662 | KU710991 |
| *C. bungeanae* | CFCC 50495^T^ | MH933621 | MH933593 | MH933497 | MH933563 | MH933529 |
| *C. bungeanae* | CFCC 50496 | MH933622 | MH933594 | MH933498 | MH933564 | MH933530 |
| *C. calamicola* | MFLUCC 15-0397 | NR_185736 | / | ON734013 | / | / |
| *C. californica* | CBS 144234^T^ | MG971935 | / | MG971645 | / | MG972083 |
| *C. carbonacea* | CFCC 89947 | KR045622 | KU710950 | KP310855 | KP310825 | KP310842 |
| *C. carpobroti* | CMW 48981^T^ | MH382812 | / | MH411212 | MH411207 | / |
| *C. celtidicola* | CFCC 50497^T^ | MH933623 | MH933595 | MH933499 | MH933566 | MH933531 |
| *C. celtidicola* | CFCC 50498 | MH933624 | MH933596 | MH933500 | MH933567 | MH933532 |
| *C. centrivillosa* | MFLUCC 16-1206^T^ | MF190122 | MF377600 | / | / | / |
| *C. centrivillosa* | MFLUCC 17-1660 | MF190123 | MF377601 | / | / | / |
| *C. ceratosperma* | CFCC 89624 | KR045645 | KU710976 | KP310860 | KR045686 | / |
| *C. ceratosperma* | CFCC 89625 | KR045646 | KU710977 | KP310861 | KR045687 | / |
| *C. ceratospermopsis* | CFCC 89626^T^ | KR045647 | KU710978 | KU710934 | KR045688 | KU711011 |
| *C. ceratospermopsis* | CFCC 89627 | KR045648 | KU710979 | KU710935 | KR045689 | KU711012 |
| *C. chrysosperma* | CFCC 89629 | KF765673 | KF765705 | / | / | / |
| *C. chrysosperma* | CFCC 89981 | MH933625 | MH933597 | MH933501 | MH933568 | MH933533 |
| *C. chrysosperma* | CFCC 89982 | KP281261 | / | KP310848 | KP310818 | KP310835 |
| *C. cinnamomea* | CFCC 53178^T^ | MK673054 | / | / | MK672970 | MK673024 |
| *C. coryli* | CFCC 53162^T^ | MN854450 | MN850751 | MN850758 | MN861120 | / |
| *C. corylina* | CFCC 54684^T^ | MW839861 | MW815937 | MW815886 | MW883969 | MW815951 |
| *C. corylina* | CFCC 54685 | MW839862 | MW815938 | MW815887 | MW883970 | MW815952 |
| *C. corylina* | CFCC 54686 | MW839863 | MW815939 | MW815888 | MW883971 | MW815953 |
| *C. corylina* | CFCC 54687 | MW839864 | MW815940 | MW815889 | MW883972 | MW815954 |
| *C. cotini* | MFLUCC 14-1050^T^ | KX430142 | KX430144 | / | / | / |
| *C. cotoneastricola* | CF 20197027 | MK673072 | MK673012 | MK672958 | MK672988 | MK673042 |
| *C. cotoneastricola* | CF 20197028 | MK673073 | MK673013 | MK672959 | MK672989 | MK673043 |
| *C. cotoneastricola* | CF 20197030 | MK673074 | MK673014 | MK672960 | MK672990 | MK673044 |
| *C. cotoneastricola* | CF 20197031^T^ | MK673075 | MK673015 | MK672961 | MK672991 | MK673045 |
| *C. curvata* | MFLUCC 15-0865^T^ | KY417728 | KY417796 | / | / | KY417694 |
| *C. curvispora* | CFCC 54000^T^ | MW839851 | MW815945 | MW815880 | MW883963 | MW815931 |
| *C. curvispora* | CFCC 54001 | MW839853 | MW815946 | MW815881 | MW883964 | MW815932 |
| *C. curvispora* | CFCC 54676 | MW839854 | MW815947 | MW815882 | MW883965 | MW815933 |
| *C. curvispora* | CFCC 54677 | MW839855 | MW815948 | MW815883 | MW883966 | MW815934 |
| *C. curvispora* | CFCC 54678 | MW839856 | MW815949 | MW815884 | MW883967 | MW815935 |
| *C. davidiana* | CXY 1350T | KM034870 | / | / | / | / |
| *C. diopuiensis* | MFLUCC 18-1419^T^ | MK912137 | / | / | / | MN685819 |
| *C. diopuiensis* | CFCC55884 | OK316819 | OK358569 | OK358471 | OK358473 | / |
| *C. diopuiensis* | CFCC55885 | OK316820 | OK358570 | OK358472 | OK358474 | / |
| *C. diopuiensis* | CFCC 56961 | ON376918 | ON390908 | ON390914 | ON390923 | ON390905 |
| *C. diopuiensis* | CFCC 56970 | ON376917 | ON390907 | ON390913 | ON390922 | ON390904 |
| *C. diopuiensis* | CFCC 56971 | ON376919 | / | ON390915 | / | ON390906 |
| *C. discotoma* | CFCC 53137^T^ | MW418404 | MW422907 | MW422919 | MW422931 | MW422897 |
| *C. discotoma* | CFCC 54368 | MW418405 | MW422908 | MW422920 | MW422932 | MW422898 |
| *C. donetzica* | MFLUCC 15-0864 | KY417729 | KY417797 | / | / | KY417695 |
| *C. donetzica* | MFLUCC 16-0574^T^ | KY417731 | KY417799 | / | / | KY417697 |
| *C. donglingensis* | CFCC 53159^T^ | MW418412 | MW422915 | MW422927 | MW422939 | MW422903 |
| *C. donglingensis* | CFCC 53160 | MW418414 | MW422917 | MW422929 | MW422941 | MW422905 |
| *C. donglingensis* | CFCC 54371 | MW418413 | MW422916 | MW422928 | MW422940 | MW422904 |
| *C. donglingensis* | CFCC 54372 | MW418415 | MW422918 | MW422930 | MW422942 | MW422906 |
| *C. elaeagni* | CFCC 89632 | KR045626 | KU710955 | KU710918 | KR045667 | KU710995 |
| *C. elaeagni* | CFCC 89633 | KF765677 | KU710956 | KU710919 | KR045668 | KU710996 |
| *C. elaeagnicola* | CFCC 52882^T^ | MK732341 | MK732347 | / | / | MK732344 |
| *C. elaeagnicola* | CFCC 52883 | MK732342 | MK732348 | / | / | MK732345 |
| *C. elaeagnicola* | CFCC 52884 | MK732343 | MK732349 | / | / | MK732346 |
| *C. ershadii* | IRAN 4197^T^ | MW295510 | / | MW394143 | / | / |
| *C. ershadii* | IRAN 4198C^T^ | MW295523 | MW824357 | MW394144 | / | MZ014510 |
| *C. erumpens* | CFCC 50022 | MH933627 | / | MH933502 | MH933569 | MH933534 |
| *C. erumpens* | MFLUCC 16-0580^T^ | KY417733 | KY417801 | / | / | KY417699 |
| *C. erumpens* | CFCC 53163 | MK673059 | MK673000 | MK672948 | MK672975 | MK673029 |
| *C. eucalypti* | CBS 144241 | MG971907 | / | MG971617 | MG971772 | MG972056 |
| *C. euonymicola* | CFCC 50499^T^ | MH933628 | MH933598 | MH933503 | MH933570 | MH933535 |
| *C. euonymicola* | CFCC 50500 | MH933629 | MH933599 | MH933504 | MH933571 | MH933536 |
| *C. euonymina* | CFCC 89993^T^ | MH933630 | MH933600 | MH933505 | MH933590 | MH933537 |
| *C. euonymina* | CFCC 89999 | MH933631 | MH933601 | MH933506 | MH933591 | MH933538 |
| *C. euonymina* | CFCC 59444 | OR826164 | OR832019 | OR832041 | / | OR831997 |
| *C. euonymina* | CFCC 59479 | OR826165 | OR832020 | OR832042 | / | OR831998 |
| *C. fengtaiensis* | CFCC 59442 | OR826166 | OR832021 | OR832043 | OR832063 | OR831999 |
| *C. fengtaiensis* | CFCC 59449^T^ | OR826167 | OR832022 | OR832044 | OR832064 | OR832000 |
| *C. fengtaiensis* | CFCC 59525 | OR826168 | OR832023 | OR832045 | OR832065 | OR832001 |
| *C. fengtaiensis* | CFCC 59526 | OR826169 | OR832024 | OR832046 | OR832066 | OR832002 |
| *C. fengtaiensis* | CFCC 59527 | OR826170 | OR832025 | OR832047 | OR832067 | OR832003 |
| *C. fraxinigena* | BBH 42442 | MF190133 | / | / | / | / |
| *C. fraxinigena* | MFLUCC 14-0868^T^ | MF190133 | / | / | / | / |
| *C. fugax* | CXY 1371 | KM034852 | / | / | KM034891 | / |
| *C. fugax* | CXY 1381 | KM034853 | / | / | KM034890 | / |
| *C. galegicola* | MFLUCC 18-1199^T^ | MK912128 | MN685820 | / | / | MN685810 |
| *C. gigalocus* | CFCC 89620^T^ | KR045628 | KU710957 | KU710920 | KR045669 | KU710997 |
| *C. gigalocus* | CFCC 89621 | KR045629 | KU710958 | KU710921 | KR045670 | KU710998 |
| *C. gigaspora* | CFCC 50014 | KR045630 | KU710959 | KU710922 | KR045671 | KU710999. |
| *C. gigaspora* | CFCC 89634^T^ | KF765671 | KU710960 | KU710923 | KR045672 | KU711000 |
| *C. globosa* | MFLU 16-2054^T^ | MT177935 | MT432212 | MT454016 | / | / |
| *C. granati* | CBS 144237^T^ | MG971799 | / | MG971514 | MG971664 | MG971949 |
| *C. haidianensis* | CFCC 54056 | MT360041 | MT363987 | MT363997 | MT364007 | MT363978 |
| *C. haidianensis* | CFCC 54057^T^ | MT360042 | MT363988 | MT363998 | MT364008 | MT363979 |
| *C. haidianensis* | CFCC 54184 | MT360043 | MT363989 | MT363999 | MT364009 | MT363980 |
| *C. haidianensis* | CFCC 59450 | OR826171 | OR832026 | OR832048 | OR832068 | OR832004 |
| *C. haidianensis* | CFCC 59475 | OR826172 | OR832027 | OR832049 | OR832069 | OR832005 |
| *C. haidianensis* | CFCC 59471 | OR826173 | OR832028 | OR832050 | OR832070 | OR832006 |
| *C. haidianensis* | CFCC 59536 | OR826174 | OR832029 | OR832051 | OR832071 | OR832007 |
| *C. hippophaës* | CFCC 89639 | KR045632 | KU710961 | KU710924 | KR045673 | KU711001 |
| *C. hippophaës* | CFCC 89640 | KF765682 | KU710962 | KP310865 | KR045674 | KF765730 |
| *C. huairouensis* | CFCC 56940 | ON188758 | OR662096 | OR662113 | OR662060 | OR662079 |
| *C. huairouensis* | CFCC 56973 | ON188759 | OR662097 | OR662114 | OR662061 | OR662080 |
| *C. huairouensis* | CFCC 57286 | ON188760 | OR662098 | OR662115 | OR662062 | OR662081 |
| *C. iranica* | IRAN 4200C^T^ | MW295652 | MW824359 | MW394146 | / | MZ014512 |
| *C. iranica* | IRAN 4628C | OM368651 | / | OM372513 | / | / |
| *C. japonica* | CFCC 89956 | KR045624 | KU710953 | KU710916 | KR045665 | KU710993 |
| *C. japonica* | CFCC 89960 | KR045625 | KU710954 | KU710917 | KR045666 | KU710994 |
| *C. joaquinensis* | CBS 144235 | MG971895 | / | MG971605 | MG971761 | MG972044 |
| *C. junipericola* | BBH 42444 | MF190126 | / | MF377579 | / | / |
| *C. junipericola* | MFLU 17-0882^T^ | MF190125 | / | MF377580 | / | / |
| *C. juniperina* | CFCC 50501^T^ | MH933632 | MH933602 | MH933507 | / | MH933539 |
| *C. juniperina* | CFCC 50502 | MH933633 | MH933603 | MH933508 | MH933572 | MH933540 |
| *C. juniperina* | CFCC 50503 | MH933634 | MH933604 | MH933509 | / | MH933541 |
| *C. kantschavelii* | CXY 1383 | KM034867 | / | / | / | / |
| *C. kantschavelii* | CXY 1386 | KM034867 | / | / | / | / |
| *C. kuanchengensis* | CFCC 52464^T^ | MK432616 | MK578076 | / | / | MK442940 |
| *C. kuanchengensis* | CFCC 52465 | MK432617 | MK578077 | / | / | MK442941 |
| *C. longispora* | CBS 144236^T^ | MG971905 | / | MG971615 | MG971764 | MG972054 |
| *C. longistiolata* | MFLUCC 16-0628 | KY417734 | KY417802 | / | / | KY417700 |
| *C. leucosperma* | CFCC 89622 | KR045616 | KU710944 | KU710911 | KR045657 | KU710988 |
| *C. leucosperma* | CFCC 89894 | KR045617 | KU710945 | KU710912 | KR045658 | KU710989 |
| *C. leucostoma* | CFCC 50023 | KR045635 | KU710964 | KU710926 | KR045676 | KU711003 |
| *C. leucostoma* | CFCC 50024 | MH933640 | MH933605 | / | MH933576 | MH933547 |
| *C. leucostoma* | CFCC 53140 | MN854445 | MN850746 | MN850753 | MN861115 | MN850760 |
| *C. leucostoma* | CFCC 53141 | MN854446 | MN850747 | MN850754 | MN861116 | MN850761 |
| *C. leucostoma* | CFCC 53156 | MN854447 | MN850748 | MN850755 | MN861117 | MN850762 |
| *C. leucostoma* | CFCC 53167 | MK673056 | MK672998 | MK672946 | MK672972 | MK673026 |
| *C. leucostoma* | CFCC 53169 | MK673080 | MK673020 | MK672966 | MK672996 | MK673050 |
| *C. leucostoma* | CFCC 53170 | MK673081 | MK673021 | MK672967 | MK672997 | MK673051 |
| *C. leucostoma* | CFCC 54680 | MW839857 | MW815955 | MW815890 | MW883973 | MW815941 |
| *C. leucostoma* | CFCC 54681 | MW839857 | MW815956 | MW815891 | MW883974 | MW815942 |
| *C. leucostoma* | CFCC 54682 | MW839857 | MW815957 | MW815892 | MW883975 | MW815943 |
| *C. leucostoma* | CFCC 54683 | MW839857 | MW815958 | MW815893 | MW883976 | MW815944 |
| *C. lumnitzericola* | MFLUCC 17-0508^T^ | MG975778 | MH253453 | / | / | MH253457 |
| *C. macropycnidia* | CBS 149338 | OP038094 | OP095265 | OP106954 | OP079909 | OP003977 |
| *C. mali* | CFCC 50028 | MH933641 | MH933606 | MH933513 | MH933577 | MH933548 |
| *C. mali* | CFCC 50029 | MH933642 | MH933607 | MH933514 | MH933578 | MH933549 |
| *C. mali* | CFCC 50030 | MH933643 | MH933608 | MH933524 | MH933579 | MH933550 |
| *C. mali* | CFCC 50031 | KR045636 | KU710965 | KU710927 | KR045677 | KU711004 |
| *C. mali* | CFCC 50044 | KR045637 | KU710966 | KU710928 | KR045678 | KU711005 |
| *C. mali-spectabilis* | CFCC 53181^T^ | MK673066 | MK673006 | MK672953 | MK672982 | MK673036 |
| *C. melnikii* | CFCC 89984 | MH933678 | MH933609 | MH933515 | MH933580 | MH933551 |
| *C. melnikii* | MFLUCC 15-0851 | KY417735 | KY417803 | / | / | KY417701 |
| *C. melnikii* | MFLUCC 16-0635 | KY417736 | KY417804 | / | / | KY417702 |
| *C. myrtagena* | CFCC 52454 | MK432614 | MK578074 | / | / | MK442938 |
| *C. myrtagena* | CFCC 52455 | MK432615 | MK578075 | / | / | MK442939 |
| *C. nivea* | MFLUCC 15-0860 | KY417737 | KY417805 | / | / | KY417703 |
| *C. nivea* | CFCC 89641 | KF765683 | KU710967 | KU710929 | KR045679 | KU711006 |
| *C. nivea* | CFCC 89643 | KF765685 | KU710968 | KP310863 | KP310829 | / |
| *C. notastroma* | NE_TFR5 | JX438632 | / | JX438543 | / | / |
| *C. notastroma* | NE_TFR8 | JX438633 | / | JX438542 | / | / |
| *C. ochracea* | CFCC 53164^T^ | MK673060 | MK673001 | MK672949 | MK672976 | MK673030 |
| *C. oleicola* | CBS 144248^T^ | MG971944 | / | MG971660 | MG971752 | MG972098 |
| *C. olivacea* | CFCC 53174 | MK673058 | MK672999 | / | MK672974 | MK673028 |
| *C. olivacea* | CFCC 53175 | MK673062 | MK673003 | / | MK672978 | MK673032 |
| *C. olivacea* | CFCC 53176^T^ | MK673068 | MK673008 | MK672955 | MK672984 | MK673038 |
| *C. olivacea* | CFCC 53177 | MK673071 | MK673011 | / | MK672987 | MK673041 |
| *C. olivarum* | UCD634-Oe,CBS 145585 | MK514094 | / | MK509030 | MK509035 | MK509025 |
| *C. olivarum* | UCD644-Oe | MK514095 | / | MK509031 | MK509036 | MK509026 |
| *C. palm* | CXY 1276 | JN402990 | / | KJ781296 | / | / |
| *C. palm* | CXY 1280^T^ | JN411939 | / | KJ781297 | / | / |
| *C. paracinnamomea* | CFCC 55453^T^ | MZ702594 | OK303515 | OK303576 | OK303643 | OK303456 |
| *C. paracinnamomea* | CFCC 55455^T^ | MZ702598 | OK303519 | OK303580 | OK303647 | OK303460 |
| *C. parakantschavelii* | MFLUCC 15-0857^T^ | KY417738 | KY417806 | / | / | KY417704 |
| *C. parakantschavelii* | MFLUCC 16-0575 | KY417739 | KY417807 | / | / | KY417705 |
| *C. parapistaciae* | CBS 144506^T^ | MG971804 | / | MG971519 | MG971669 | MG971954 |
| *C. parasitica* | MFLUCC 15-0507^T^ | KY417740 | KY417808 | / | / | KY417706 |
| *C. parasitica* | XJAU 2542-1 | MH798884 | / | MH813452 | / | / |
| *C. parasitica* | CFCC 53171 | MK673061 | MK673002 | MK672950 | MK672977 | MK673031 |
| *C. parasitica* | CFCC 53172 | MK673069 | MK673009 | MK672956 | MK672985 | MK673039 |
| *C. parasitica* | CFCC 53173 | MK673070 | MK673010 | MK672957 | MK672986 | MK673040 |
| *C. paratranslucens* | MFLUCC 15-0506^T^ | KY417741 | KY417809 | / | / | KY417707 |
| *C. paratranslucens* | MFLUCC 16-0627 | KY417742 | KY417810 | / | / | KY417708 |
| *C. paraplurivora* | FDS-439 | OL640182 | / | OL631589 | / | OL631586 |
| *C. paraplurivora* | FDS-564 | OL640183 | / | OL631590 | / | OL631587 |
| *C. paraplurivora* | FDS-623 | OL640181 | / | OL631591 | / | OL631588 |
| *C. phialidica* | MFLUCC 17-2498 | MT177932 | MT432209 | MT454014 | / | / |
| *C. piceae* | CFCC 52841^T^ | MH820398 | MH820395 | MH820402 | MH820387 | MH820406 |
| *C. piceae* | CFCC 52842 | MH820399 | MH820396 | MH820403 | MH820388 | MH820407 |
| *C. pinea* | CFCC 59521^T^ | OR826181 | OR832036 | OR832058 | OR832078 | OR832014 |
| *C. pinea* | CFCC 59522 | OR826182 | OR832037 | OR832059 | OR832079 | OR832015 |
| *C. pinea* | CFCC 59523 | OR826183 | OR832038 | OR832060 | OR832080 | OR832016 |
| *C. pinea* | CFCC 59524 | OR826184 | OR832039 | OR832061 | OR832081 | OR832017 |
| 1. *pingbianensis* | MFLUCC 18-1204^T^ | MK912135 | MN685826 | / | / | MN685817 |
| *C. pistaciae* | CBS 144238^T^ | MG971802 | / | MG971517 | MG971667 | MG971952 |
| *C. platanicola* | MFLU 17-0327 | MH253451 | MH253450 | / | / | MH253449 |
| *C. platyclada* | CFCC 50504^T^ | MH933645 | MH933610 | MH933516 | MH933581 | MH933552 |
| *C. platyclada* | CFCC 50505 | MH933646 | MH933611 | MH933517 | MH933582 | MH933553 |
| *C. platyclada* | CFCC 50506 | MH933647 | MH933612 | MH933518 | MH933583 | MH933554 |
| *C. platycladicola* | CFCC 50038^T^ | KT222840 | MH933613 | MH933519 | MH933584 | MH933555 |
| *C. platycladicola* | CFCC 50039 | KR045642 | KU710973 | KU710931 | KR045683 | KU711008 |
| *C. plurivora* | CBS 144239^T^ | MG971861 | / | MG971572 | MG971726 | MG972010 |
| *C. populicola* | CBS 144240 | MG971891 | / | MG971601 | MG971757 | MG972040 |
| *C. populina* | CFCC 89644^T^ | KF765686 | KU710969 | KU710930 | KR045681 | KU711007 |
| *C. populinopsis* | CFCC 50032^T^ | MH933648 | MH933614 | MH933520 | MH933585 | MH933556 |
| *C. populinopsis* | CFCC 50033 | MH933649 | MH933615 | MH933521 | MH933586 | MH933557 |
| *C. predappioensis* | MFLUCC 17-2458^T^ | MG873484 | / | / | / | / |
| *C. prunicola* | MFLU 17-0995^T^ | MG742350 | MG742352 | / | / | MG742353 |
| *C. pruni-mume* | CFCC 53179 | MK673057 | / | MK672947 | MK672973 | MK673027 |
| *C. pruni-mume* | CFCC 53180^T^ | MK673067 | MK673007 | MK672954 | MK672983 | MK673037 |
| *C. prunina* | CFCC 58997 | OR578808 | / | / | OR662077 | / |
| *C. prunina* | CFCC 58998 | OR578809 | / | / | OR662078 | / |
| *C. pruinopsis* | CFCC 50034^T^ | KP281259 | KU710970 | KP310849 | KP310819 | KP310836 |
| *C. pruinopsis* | CFCC 50035 | KP281260 | KU710971 | KP310850 | KP310820 | KP310837 |
| *C. pruinopsis* | CFCC 53153 | MN854451 | MN850752 | MN850759 | MN861121 | MN850763 |
| *C. pruinosa* | CFCC 50036 | KP310800 | / | KP310845 | KP310815 | KP310832 |
| *C. pruinosa* | CFCC 50037 | MH933650 | / | MH933522 | MH933589 | MH933558 |
| *C. pubescentis* | MFLUCC 18-1201^T^ | MK912130 | MN685821 | / | / | MN685812 |
| *C. punicae* | CBS 144244 | MG971943 | / | MG971654 | MG971798 | MG972091 |
| *C. quercicola* | MFLU 17-0881 | MF190128 | / | / | / | / |
| *C. quercicola* | MFLUCC 14-0867^T^ | MF190129 | / | / | / | / |
| ***C. qujingensis*** | **GMBCC1004** | **PP829298** | **PP839459** | **PP839466** | **PP839450** | **PP850108** |
| ***C. qujingensis*** | **ZHKUCC 23-0978^T^** | **PP829299** | **PP839460** | **PP839467** | **PP839451** | **PP850109** |
| ***C. qujingensis*** | **ZHKUCC 23-0979** | **PP829300** | **PP839461** | **PP839468** | **PP839452** | **PP850110** |
| *C. ribis* | CFCC 50026 | KP281267 | KU710972 | KP310856 | KP310826 | KP310843 |
| *C. ribis* | CFCC 50027 | KP281268 | / | KP310857 | KP310827 | KP310844 |
| *C. rosae* | MFLU 17-0885 | MF190131 | / | / | / | / |
| *C. rosicola* | CF 20197024^T^ | MK673079 | MK673019 | MK672965 | MK672995 | MK673049 |
| *C. rosigena* | MFLUCC 18-0921^T^ | MN879872 | / | / | / | / |
| *C. rostrata* | CFCC 89909 | KR045643 | KU710974 | KU710932 | KR045684 | KU711009 |
| *C. rostrata* | CFCC 89910 | KR045644 | KU710975 | KU710933 | / | KU711010 |
| *C. rusanovii* | MFLUCC 15-0853 | KY417743 | KY417811 | / | / | KY417709 |
| *C. rusanovii* | MFLUCC 15-0854^T^ | KY417744 | KY417812 | / | / | KY417710 |
| *C. salicacearum* | MFLUCC 15-0509 | KY417746 | KY417814 | / | / | KY417712 |
| *C. salicacearum* | MFLUCC 15-0861 | KY417745 | KY417813 | / | / | KY417711 |
| *C. salicacearum* | MFLUCC 16-0587 | KY417748 | KY417816 | / | / | KY417714 |
| *C. salicacearum* | MFLUCC 16-0576 | KY417748 | KY417815 | / | / | KY417713 |
| *C. salicicola* | MFLUCC 14-1052^T^ | KU982636 | / | / | / | KU982637 |
| *C. salicicola* | MFLUCC 15-0866 | KY417749 | KY417817 | / | / | KY417715 |
| *C. salicina* | MFLUCC 15-0862 | KY417750 | KY417818 | / | / | KY417716 |
| *C. salicina* | MFLUCC 16-0637 | KY417751 | KY417819 | / | / | KY417717 |
| *C. schulzeri* | CFCC 50040 | KR045649 | KU710980 | KU710936 | KR045690 | KU711013 |
| *C. schulzeri* | CFCC 50042 | KR045650 | KU710981 | KU710937 | KR045691 | KU711014 |
| ***C. schulzeri*** | **GMBCC1005** | **PP829301** | **PP839462** | **PP839469** | **PP839453** | **PP850111** |
| ***C. schulzeri*** | **ZHKUCC 23-0980** | **PP829302** | **PP839463** | **PP839470** | **PP839454** | **PP850112** |
| *C. sibiraeae* | CFCC 50045^T^ | KR045651 | KU710982 | KU710938 | KR045692 | KU711015 |
| *C. sibiraeae* | CFCC 50046 | KR045652 | KU710983 | KU710939 | KR045693 | KU711016 |
| *C. sophorae* | CFCC 50047 | KR045653 | KU710984 | KU710940 | KR045694 | KU711017 |
| *C. sophorae* | CFCC 50048 | MH820401 | MH820397 | MH820405 | MH820390 | MH820409 |
| *C. sophorae* | CFCC 89598 | KR045654 | KU710985 | KU710941 | KR045695 | KU711018 |
| *C. sophoricola* | CFCC 89596 | KR045656 | KU710987 | KU710943 | KR045697 | KU711020 |
| *C. sophoricola* | CFCC 89595^T^ | KR045655 | KU710986 | KU710942 | KR045696 | KU711019 |
| *C. sophoriopsis* | CFCC 55469 | MZ702583 | OK303504 | OK303565 | OK303632 | OK303445 |
| *C. sophoriopsis* | CFCC 89600 | KR045623 | KU710951 | KU710915 | KP310817 | KU710992 |
| *C. sorbariae* | CFCC 59443 | OR826175 | OR832030 | OR832052 | OR832072 | OR832008 |
| *C. sorbariae* | CFCC 59445^T^ | OR826176 | OR832031 | OR832053 | OR832073 | OR832009 |
| *C. sorbariae* | CFCC 59529 | OR826177 | OR832032 | OR832054 | OR832074 | OR832010 |
| *C. sorbariae* | CFCC 59530 | OR826178 | OR832033 | OR832055 | OR832075 | OR832011 |
| *C. sorbi* | MFLUCC 16-0631^T^ | KY417752 | KY417820 | / | / | KY417718 |
| *C. sorbicola* | MFLUCC 16-0584^T^ | KY417755 | KY417823 | / | / | KY417721 |
| *C. sorbicola* | MFLUCC 16-0633 | KY417758 | KY417826 | / | / | KY417724 |
| *C. sorbina* | CF 20197660^T^ | MK673052 | / | MK672943 | MK672968 | MK673022 |
| *C. spiraeae* | CFCC 50049^T^ | MG707859 | MG708199 | / | / | MG708196 |
| *C. spiraeae* | CFCC 50050 | MG707860 | MG708200 | / | / | MG708197 |
| *C. spiraeicola* | CFCC 53138^T^ | MN854448 | MN850749 | MN850756 | MN861118 | / |
| *C. spiraeicola* | CFCC 53139 | MN854449 | MN850750 | MN850757 | MN861119 | / |
| *C. tamaricicola* | CFCC 50507 | MH933651 | MH933616 | MH933525 | MH933587 | MH933559 |
| *C. tamaricicola* | CFCC 50508^T^ | MH933652 | MH933617 | MH933523 | MH933588 | MH933560 |
| *C. tanaitica* | MFLUCC 14-1057^T^ | KT459411 | / | / | / | KT459413 |
| *C. thailandica* | MFLUCC 17-0262^T^ | MG975776 | MH253455 | / | / | MH253459 |
| *C. thailandica* | MFLUCC 17-0263^T^ | MG975777 | MH253456 | / | / | MH253460 |
| *C. tibetensis* | CF 20197026 | MK673076 | MK673016 | MK672962 | MK672992 | MK673046 |
| *C. tibetensis* | CF 20197029 | MK673077 | MK673017 | MK672963 | MK672993 | MK673047 |
| *C. tibetensis* | CF 20197032^T^ | MK673078 | MK673018 | MK672964 | MK672994 | MK673048 |
| *C. tibouchinae* | CPC 26333^T^ | KX228284 | / | / | / | / |
| *C. translucens* | CXY 1351 | KM034874 | / | / | KM034895 | / |
| *C. translucens* | CXY 1359 | KM034871 | / | / | KM034894 | / |
| *C. ulmi* | MFLUCC 15-0863^T^ | KY417759 | / | / | / | / |
| *C. verrucosa* | CFCC 53157^T^ | MW418408 | MW422911 | MW422923 | MW422935 | / |
| *C. verrucosa* | CFCC 53158 | MW418410 | MW422913 | MW422925 | MW422937 | MW422901 |
| *C. verrucosa* | CFCC 54369 | MW418409 | MW422912 | MW422924 | MW422936 | / |
| *C. verrucosa* | CFCC 54370 | MW418411 | MW422914 | MW422926 | MW422938 | MW422902 |
| *C. vinacea* | CBS 141585^T^ | KX256256 | / | KX256277 | KX256235 | / |
| *C. viridistroma* | CBS 202.36^T^ | MN172408 | / | MN271853 | / | / |
| *C. viticola* | Cyt2 | KX256238 | / | KX256259 | KX256217 | / |
| *C. viticola* | CBS 141586^T^ | KX256239 | / | KX256260 | KX256218 | / |
| *C. xinjiangensis* | CFCC 53182 | MK673064 | MK673004 | MK672951 | MK672980 | MK673034 |
| *C. xinjiangensis* | CFCC 53183^T^ | MK673065 | MK673005 | MK672952 | MK672981 | MK673035 |
| *C. xinglongensis* | CFCC 52458^T^ | MK432622 | MK578082 | / | / | MK442946 |
| *C. xinglongensis* | CFCC 52459 | MK432623 | MK578083 | / | / | MK442947 |
| *C. xylocarpi* | MFLUCC 17-0251^T^ | MG975775 | MH253454 | / | / | MH253458 |
| *C. yakimana* | CBS 149297 | OM976602 | ON045093 | ON012569 | ON086750 | ON012555 |
| *C. yakimana* | CBS 149298 | OM976603 | ON045094 | ON012570 | ON086751 | ON012556 |
| *C. zhaitangensis* | CFCC 56227^T^ | OQ344750 | OQ398789 | OQ410623 | OQ398733 | OQ398760 |
| *C. zhaitangensis* | CFCC 57537 | OQ344751 | OQ398790 | OQ410624 | OQ398734 | OQ398761 |
| *Diaporthe vaccinii* | CBS 160.32 | KC343228 | / | KC343954 | KC344196 | JQ807297 |

**TABLE S3:** The names, isolate numbers, and corresponding GenBank accession numbers of the taxa used in Figure 2. The taxa produced in this study are indicated in bold, and the type strains are indicated in bold with “T”. “/”: no data available in GenBank.

| **Species name** | **Isolate No.** | **GenBank accession No.** | |
| --- | --- | --- | --- |
|  |  | **ITS** | ***tub*2** |
| *Allocryptovalsa aceris* | KUMCC:21-0085 | MZ727001 | OK043823 |
| *A. aquilariae* | KUNCC 22-10819^T^ | OP454035 | OP572197 |
| *A. aquilariae* | KUNCC 22-12389 | OP456373 | OP572198 |
| *A. castaneae* | CFCC 52428T | MW632945 | MW656393 |
| ***A. castaneae*** | **ZHKUCC 23-0981** | **PP829303** | **PP839455** |
| ***A. castaneae*** | **ZHKUCC 23-0982** | **PP829304** | **PP839456** |
| *A. castaneae* | CFCC 52427 | MW632944 | MW656392 |
| *A. castaneicola* | CFCC 52432T | MW632947 | MW656395 |
| *A. cryptovalsoidea* | HVFIG02T | HQ692573 | HQ692524 |
| *A. cryptovalsoidea* | HVFIG05 | HQ692574 | HQ692525 |
| *A. elaeidis* | MFLUCC 15-0707 | MN308410 | MN340296 |
| *A. polyspora* | MFLUCC 17-0364^T^ | MF959500 | MG334556 |
| *A. rabenhorstii* | WA07CO | HQ692620 | HQ692522 |
| *A. rabenhorstii* | WA08CB | HQ692619 | HQ692523 |
| *A. sichuanensis* | HKAS 107017 | MW240633 | MW775592 |
| *A. truncata* | PUFNI17639T | MK990279 | / |
| *A. xishuangbanica* | KUMCC 21-0830^T^ | ON041128 | ON081498 |
| *A. xishuangbanica* | KUMCC 21-0829 | ON041131 | ON081501 |
| *Eutypella australiensis* | STEU_8248 | MF359657 | MF359691 |

**TABLE S4:** The names, isolate numbers, and corresponding GenBank accession numbers of the taxa used in Figure 3. The taxa produced in this study are indicated in bold, and the type strains are indicated in bold with “T”. “/”: no data available in GenBank.

| **Species** | **Isolate No.** | **GenBank accession No.** | |
| --- | --- | --- | --- |
|  |  | **ITS** | **LSU** |
| *Aureobasidium acericola* | CDH 2020-10 | MT863788 | MT863787 |
| *A. aerium* | CFCC 50324 | ON007058 | ON007081 |
| *A. bupleuri* | CBS 131304 | KT693738 | / |
| *A. castanea* | CFCC 54591 | NR_177551 | MW364275 |
| *A. caulivorum* | CBS 242.64 | FJ150871 | FJ150944 |
| *A. faidherbiae* | CBS 149677 | OQ628485 | OQ629067 |
| *A. insectorum* | KCL139 | OP856707 | OP857208 |
| *A. intercalariosporum* | MQL9-100 | OP856704 | OP857205 |
| *A. iranianum* | CCTU 268 | NR_137598 | NG_057049 |
| *A. khasianum* | NFCCI 4275 | MH188305 | MH188306 |
| *A. leucospermi* | CBS 130593 | NR_156246 | MH877257 |
| *A. mangrovei* | IBRC M 30265^T^ | NR_174637 | NG_078639 |
| *A. melanogenum* | CBS 105.22 | NR_159598 | NG_056960 |
| *A. microstictum* | CBS 342.66 | KT693743 | FJ150945 |
| *A. microstictum* | CBS 114.64 | KT693744 | KT693986 |
| *A. microtermitis* | GTS2.7 | MW276135 | MW276136 |
| *A. motuoense* | E31-1 | OP856709 | OP857210 |
| *A. motuoense* | E26-4 | OP856708 | OP857209 |
| *A. namibiae* | CBS 147.97 | FJ150875 | FJ150937 |
| *A. pini* | CFCC 52778 | MK184533 | MK184535 |
| *A. planticola* | MDSC-10 | OP856711 | OP857212 |
| *A. proteae* | CBS 114273 | JN712491 | JN712557 |
| *A. proteae* | CPC 13701 | JN712490 | JN712556 |
| *A. pullulans* | CBS 584.75 | FJ150906 | FJ150942 |
| *A. pullulans* | CBS 146.30 | FJ150902 | FJ150916 |
| ***A. pullulans*** | **ZHKUCC 23-0983** | **PP829305** | **PP842127** |
| ***A. pullulans*** | **GMBCC1006** | **PP829306** | **PP842128** |
| *A. subglaciale* | EXF-2481 | FJ150895 | FJ150913 |
| *A. thailandense* | NRRL 58539^T^ | Jx462674 | / |
| *A. thailandense* | NRRL 58543 | Jx462675 | / |
| *A. tremulum* | UN_1 | MK503657 | MK503660 |
| *A. welwitschiae* | CBS 149676 | NR_189528 | NG_242108 |
| *A. xishuangbannaense* | KUMCC 21-0703 | ON426835 | OP363258 |
| *Sydouia polyspora* | CBS 750.71 | MH860332 | MH872085 |

**TABLE S5:** The names, isolate numbers, and corresponding GenBank accession numbers of the taxa used in Figure 4. The taxa produced in this study are indicated in bold, and the type strains are indicated in bold with “T”. “/”: no data available in GenBank.

| **Species name** | **Isolate No.** | **GenBank Accession No** | | | |
| --- | --- | --- | --- | --- | --- |
|  |  | **ITS** | **LSU** | ***rpb*2** | **tub** |
| *Annulohypoxylon moriforme* | CBS 123579 | KX376321 | KY610425 | KY624289 | KX271261 |
| *A. truncatum* | CBS 140778^T^ | KX376329 | KY610419 | KY624277 | KX376352 |
| *Annulohypoxylon annulatum* | CBS 140775^T^ | KU604559 | KY610418 | KY624263 | KX376353 |
| *Biscogniauxia nummularia* | MUCL 51395 | KY610382 | KY610427 | KY624236 | KX271241 |
| *Camporesia sambuci* | MFLU 15-3905^T^ | KU746392 | KU746394 | KU746390 | / |
| *Daldinia dennisii* | CBS 114741^T^ | JX658477 | KY610435 | KY624244 | KC977262 |
| *D. petriniae* | MUCL 49214^T^ | JX658512 | KY610439 | KY624248 | KC977261 |
| *Hypomontagnella barbarensis* | STMA 14081^T^ | MK131720 | MK131718 | MK135891 | MK135893 |
| *H. monticulosa* | MUCL 54604^T^ | KY610404 | KY610487 | KY624305 | KX271273 |
| *H.submonticulosa* | CBS 115280 | KC968923 | KY610457 | KY624226 | KC977267 |
| *Hypoxylon addis* | MUCL 52797^T^ | KC968931 | ON954141 | OP251037 | KC977287 |
| *H. aeneipigmentatum* | BRFM 3043 | OR415256 | / | / | / |
| *H. anthochroum* | YMJ 9 | JN660819 | / | / | AY951703 |
| *H. asterostomum* | BRFM 3112 | OR415311 | / | / | / |
| *H. atrovinosum* | BRFM 3045 | OR415327 | / | / | / |
| *H. atypicum* | BRFM 3127 | OR451662 | / | / | / |
| *H. aveirense* | MUM 19.40^T^ | MN053021 | ON954142 | OP251028 | MN066636 |
| *H. avellanicolor* | BRFM 3110 | OR415328 | / | / | / |
| *H. arawakianum* | MJF 13020^T^ | OR451660 | / | / | / |
| *H. aurantium* | MFLU 16-1202^T^ | MN047114 | MN017878 | MN077081 | / |
| *H. baihualingense* | FCATAS 477^T^ | MG490190 | / | / | MH790276 |
| *H. baruense* | UCH9545^T^ | MN056428 | / | / | MK908142 |
| *H. begae* | YMJ 215 | JN660820 | / | / | AY951704 |
| *H. bellicolor* | UCH9543^T^ | MN056425 | / | / | MK908139 |
| *H. bimaculatum* | BRFM 3487 | OR415331 | / | / | / |
| *H. brevisporum* | YMJ 36 | JN660821 | / | / | AY951705 |
| *H. blackburniae* | BRIP 72467b | NR_182618 | NG_149119 | / | / |
| *H. calileguense* | STMA 14059^T^ | KU604566 | / | / | KU604579 |
| *H. carneum* | MUCL 54177 | KY610400 | KY610480 | KY624297 | KX271270 |
| *H. cercidicola* | CBS 119009 | KC968908 | KY610444 | KY624254 | KU684189 |
| *H. chionostomum* | STMA 14060 | KU604563 | ON954144 | OP251030 | ON813072 |
| *H. chrysalidosporum* | FCATAS2710^T^ | OL467294 | OL615106 | OL584222 | OL584229 |
| *H. cinnabarinum* | BCRC 34055 | JN979409 | / | / | AY951708 |
| *H. cinnabarinum* | UCH9546 | MN056429 | / | / | MK908143 |
| *H. cocois* | MFLUCC 23-0137^T^ | OR438423 | OR438883 | OR634960 | OR538093 |
| *H. confusum* | BRFM 3475 | OR415334 | / | / | / |
| *H. crocopeplum* | CBS 119004 | KC968907 | KY610445 | KY624255 | KC977268 |
| *H. cyclobalanopsidis* | FCATAS2714^T^ | OL467298 | OL615108 | OL584225 | OL584232 |
| *H. damuense* | XZ207 | ON075427 | ON075433 | / | ON093245 |
| *H. delonicis* | MFLU 16-1031^T^ | MT215503 | MT386008 | / | MT212215 |
| *H. dieckmannii* | YMJ 89041203 | JN979413 | / | / | AY951713 |
| *H. diperithecium* | FCATAS 4226^T^ | ON178671 | ON350864 | ON365561 | ON365565 |
| *H. duranii* | YMJ 85 | JN979414 | / | / | AY951714 |
| *H. erythrostroma* | YMJ 90080602 | JN979416 | / | / | AY951716 |
| *H. eurasiaticum* | MUCL 57720^T^ | MW367851 | / | MW373852 | MW373861 |
| *H. fendleri* | DSM 107927 | MK287533 | MK287545 | MK287558 | MK287571 |
| *H. ferrugineum* | CBS 141259 | KX090079 | / | / | KX090080 |
| *H. flavoargillaceum* | STMA 14062 | KU604577 | / | / | KU159532 |
| *H. florendophyticum* | GUCC 193025^T^ | ON791190 | ON791224 | ON815920 | ON815991 |
| *H. fragiforme* | MUCL 51264^T^ | KM186294 | KM186295 | KM186296 | KM186293 |
| *H. fraxinophilum* | MUCL 54176^T^ | KC968938 | / | / | KC977301 |
| *H. fulvosulphureum* | MFLUCC 13-0589^T^ | KP401576 | / | / | KP401584 |
| *H. fuscopurpureum* | BCRC 34067 | JN979421 | / | / | AY951721 |
| *H. fuscoides* | MUCL 52670^T^ | ON792789 | ON954145 | OP251038 | ON813076 |
| *H. fuscum* | CBS 113049^T^ | KY610401 | KY610482 | KY624299 | KX271271 |
| *H. gibriacense* | MUCL 52698^T^ | KC968930 | / | / | / |
| *H. greiderae* | BRIP 72533b | OP599623 | OP598062 | / | / |
| *H. griseobrunneum* | CBS 331.73^T^ | KY610402 | MH872399 | KY624300 | KC977303 |
| *H. guilanense* | MUCL 57726^T^ | MT214997 | MT214992 | MT212235 | MT212239 |
| *H. guiyangense* | KUNCC23-15543^T^ | PP584751 | PP584824 | / | PP951417 |
| *H. guizhouense* | KUNCC23-15544^T^ | PP584753 | PP584826 | PP993509 | PP951419 |
| *H. haematostroma* | MUCL 53301^T^ | KC968911 | KY610484 | KY624301 | KC977291 |
| *H. hainanense* | FCATAS2712^T^ | OL467296 | OL616132 | OL584224 | OL584231 |
| *H. hainanense* | FCATAS2713 | OL467297 | / | / | / |
| *H. hepaticolor* | ILLS00121426^T^ | MT799854 | MT799853 | / | / |
| *H. hinnuleum* | MUCL 3621^T^ | MK287537 | MK287549 | MK287562 | MK287575 |
| *H. hinnuleum* | DSM:107932 | MK287535 | MK287547 | MK287560 | MK287573 |
| *H. hinnuleum* | DSM:107926 | MK287532 | MK287544 | MK287557 | MK287570 |
| *H. hongheensis* | HKAS 122663^T^ | OM001336 | OM001339 | ON392009 | ON468656 |
| *H. howeanum* | MUCL 47599 | AM749928 | KY610448 | KY624258 | KC977277 |
| *H. hypomiltum* | MUCL 51845 | KY610403 | KY610449 | KY624302 | KX271249 |
| *H. inaequale* | KUNCC 22-10798 | ON329812 | ON329815 | / | OQ652093 |
| *H. inusitatum* | BRFM 3369 | OR473657 | / | / | / |
| *H. invadens* | MUCL 51475^T^ | MT809133 | MT809132 | MT813037 | MT813038 |
| *H. isabellinum* | STMA10247^T^ | KC968935 | / | / | KC977295 |
| *H. jaklitschii* | CBS 138916^T^ | KM610290 | / | / | KM610304 |
| *H. jecorinum* | YMJ 39 | JN979429 | / | / | AY951731 |
| *H. jianfengense* | FCATAS845^T^ | MW984546 | MZ029707 | MZ047260 | MZ047264 |
| *H. larissae* | FCATAS844^T^ | MW984548 | MZ029706 | MZ047258 | MZ047262 |
| *H. laschii* | MUCL 52796 | JX658525 | ON954147 | OP251027 | ON813075 |
| *H. lateripigmentum* | MUCL 53304^T^ | KC968933 | KY610486 | KY624304 | KC977290 |
| *H. lechatii* | MUCL 54609 | KF923407 | ON954148 | OP251033 | KF923405 |
| *H. lenormandii* | CBS 135869 | KY610390 | KY610453 | KY624262 | KM610295 |
| *H. lienhwacheense* | MFLUCC 14-1231 | KU604558 | MK287550 | MK287563 | KU159522 |
| *H. lignicola* | MFLUCC 16-0926^T^ | MK828609 | MK835808 | MN156534 | / |
| *H. lilloi* | STMA 14142 | KU604574 | / | / | KU159537 |
| *H. liviae* | CBS 115282^T^ | KC968922 | / | / | KC977265 |
| *H. lividicolor* | BCRC 34076^T^ | JN979432 | / | / | AY951734 |
| *H. lividipigmentum* | BCRC 34077 | JN979433 | / | / | AY951735 |
| *H. judithwrightiae* | BRIP 72744d^T^ | OR673888 | OR673897 | / | / |
| *H. macrocarpum* | CBS 119012 | ON792785 | ON954151 | OP251034 | ON813071 |
| ***H. malongense*** | **ZHKUCC 23-0984^T^** | **PP829307** | **PP842129** | **PP839464** | **PP839457** |
| ***H. malongense*** | **GMBCC1007** | **PP829308** | **PP842130** | **PP839465** | **PP839458** |
| *H. mangrovei* | MFLU 18-0559^T^ | MN047116 | MN017880 | / | MN077053 |
| *H. montagnei* | GYJF 19197 | OR477330 | / | / | / |
| *H. munkii* | MUCL 53315 | KC968912 | / | / | KC977294 |
| *H. musceum* | MUCL 53765 | KC968926 | KY610488 | KY624306 | KC977280 |
| *H. neosublenormandii* | MFLUCC 11-0618^T^ | KU940157 | KU863145 | / | / |
| *H. notatum* | YMJ 250 | JQ009305 | / | / | AY951739 |
| *H. ochraceum* | MUCL 54625^T^ | KC968937 | / | KY624271 | KC977300 |
| *H. olivaceopigmentum* | DSM 10792^T^ | MK287530 | MK287542 | MK287555 | MK287568 |
| *H. okazakiorum* | MST F22864 | PQ066523 | PQ060434 | / | / |
| *H. pallidobrunneum* | BRFM 3479 | OR416203 | / | / | / |
| *H. papillatum* | ATCC 58729^T^ | KC968919 | KY610454 | KY624223 | KC977258 |
| *H. perforatum* | CBS 115281 | KY610391 | KY610455 | KY624224 | KX271250 |
| *H. petriniae* | CBS 114746^T^ | KY610405 | KY610491 | KY624279 | KX271274 |
| *H. phuphaphetense* | TBRC 16277^T^ | OP856538 | OP856528 | / | / |
| *H. pilgerianum* | STMA 13455 | KY610412 | KY610412 | KY624308 | KY624315 |
| *H. polyporoideum* | BCRC 34088 | JQ009311 | / | / | AY951747 |
| *H. porphyreum* | CBS 119022 | KC968921 | KY610456 | KY624225 | KC977264 |
| *H. pseudofendleri* | MFLUCC 11-0639 | KU940156 | KU863144 | / | / |
| *H. pulicicidum* | CBS 122622^T^ | JX183075 | KY610492 | KY624280 | JX183072 |
| *H. purpureobadium* | BRFM 3041 | OR451667 | / | / | / |
| *H. rickii* | MUCL 53309^T^ | KC968932 | KY610416 | KY624281 | KC977288 |
| *H. rosicaulendophyticum* | GUCC 191108.1^T^ | ON791192 | ON791226 | ON815922 | ON815993 |
| *H. rubiginosum* | MUCL 52887^T^ | KC477232 | KY610469 | KY624266 | KY624311 |
| *H. samuelsii* | MUCL 51843^T^ | KC968916 | KY610466 | KY624269 | KC977286 |
| *H. saulense* | BRFM 3042 | OR451669 | / | / | / |
| *H. setariae* | BRIP 70578a^T^ | PP663691 | PP663692 | PP682340 | PP682341 |
| *H. shearii* | YMJ 29 | EF026142 | / | / | AY951753 |
| *H. spegazzinianum* | STMA 14082^T^ | KU604573 | / | / | KU604582 |
| *H. sporistriatatunicum* | UCH9542^T^ | MN056426 | / | / | MK908140 |
| *H. sporoboli* | BRIP 68818a^T^ | OM417259 | OM333573 | ON624214 | ON995128 |
| *H. subgilvum* | YMJ 88113007 | JQ009315 | / | / | AY951755 |
| *H. sublenormandii* | JF 13026^T^ | KM610291 | / | / | KM610303 |
| *H. subrutiloides* | F 202416 | FJ185304 | / | / | FJ185281 |
| *H. subticinense* | MUCL 53752 | KC968913 | ON954152 | / | KC977297 |
| *H. szostakii* | BRIP 72527b | OP599624 | OP598063 | / | / |
| *H. teeravasati* | PUFD 4 | KY863509 | MF385274 | MG986895 | MG986894 |
| *H. texense* | DSM 107933^T^ | MK287536 | MK287548 | MK287561 | MK287574 |
| *H. tibeticum* | FCATAS4022^T^ | OR654146 | OR654303 | ON254302 | ON230084 |
| *H. ticinense* | CBS 115271 | JQ009317 | KY610471 | KY624272 | AY951757 |
| *H. trugodes* | MUCL 54794^T^ | KF234422 | NG066380 | KY624282 | KF300548 |
| *H. ulmophilum* | YMJ 350 | JQ009320 | / | / | AY951760 |
| *H. vinosopulvinatum* | HAST 90080707^T^ | JQ009321 | / | / | AY951761 |
| *H. vogesiacum* | CBS 115273 | KC968920 | KY610417 | KY624283 | KX271275 |
| *H. unimaculatum* | BRFM 3477 | OR420072 | / | / | / |
| *H. wujiangensis* | GMBC0213^T^ | MT568854 | MT568853 | MT585802 | MT572481 |
| *H. wuzhishanense* | FCATAS2708 | OL467292 | OL615104 | OL584220 | OL584227 |
| *H. zangii* | XZ29 | ON075423 | ON075429 | ON093247 | ON093241 |
| *H. zhaotongense* | GMBCC1168^T^ | OP597690 | OP598100 | OP615662 | OP615660 |
| *Jackrogersella cohaerens* | CBS 119126 | KY610396 | KY610497 | KY624270 | KY624314 |
| *J. multiformis* | CBS 119016^T^ | KC477234 | KY610473 | KY624290 | KX271262 |
| *Pyrenopolyporus hunteri* | MUCL 52673^T^ | KY610421 | KY610472 | KY624309 | KU159530 |
| *P. laminosus* | MUCL 53305^T^ | KC968934 | KY610485 | KY624303 | KC977292 |
| *P. nicaraguensis* | CBS 117739 | AM749922 | KY610489 | KY624307 | KC977272 |
| *Rhopalostroma angolense* | CBS 126414 | KY610420 | KY610459 | KY624228 | KX271277 |
| *Thamnomyces dendroidea* | CBS 123578^T^ | FN428831 | KY610467 | KY624232 | KY624313 |
| *Xylaria arbuscula* | CBS 126415 | KY610394 | KY610463 | KY624287 | KX271257 |
| *X. hypoxylon* | CBS 122620^T^ | KY610407 | KY610495 | KY624231 | KX271279 |
